# A Multi-Kingdom Study Reveals the Plasticity of the Rumen Microbiota in Response to a Shift From Non-grazing to Grazing Diets in Sheep

**DOI:** 10.3389/fmicb.2019.00122

**Published:** 2019-02-11

**Authors:** Alejandro Belanche, Alison H. Kingston-Smith, Gareth W. Griffith, Charles J. Newbold

**Affiliations:** ^1^Institute of Biological, Environmental and Rural Sciences, Aberystwyth University, Aberystwyth, United Kingdom; ^2^Estación Experimental del Zaidín (CSIC), Granada, Spain; ^3^Scotland's Rural College, Edinburgh, United Kingdom

**Keywords:** core microbes, grazing, network analysis, rumen microbiota, taxa abundance

## Abstract

Increasing feed efficiency is a key target in ruminant science which requires a better understanding of rumen microbiota. This study investigated the effect of a shift from a non-grazing to a grazing diet on the rumen bacterial, methanogenic archaea, fungal, and protozoal communities. A systems biology approach based on a description of the community structure, core microbiota, network analysis, and taxon abundance linked to the rumen fermentation was used to explore the benefits of increasing depth of the community analysis. A total of 24 sheep were fed ryegrass hay supplemented with concentrate (CON) and subsequently ryegrass pasture (PAS) following a straight through experimental design. Results showed that concentrate supplementation in CON-fed animals (mainly starch) promoted a simplified rumen microbiota in terms of network density and bacterial, methanogen and fungal species richness which favored the proliferation of amylolytic microbes and VFA production (+48%), but led to a lower (ca. 4-fold) ammonia concentration making the N availability a limiting factor certain microbes. The adaptation process from the CON to the PAS diet consisted on an increase in the microbial concentration (biomass of bacteria, methanogens, and protozoa), diversity (+221, +3, and +21 OTUs for bacteria, methanogens, and fungi, respectively), microbial network complexity (+18 nodes and +86 edges) and in the abundance of key microbes involved in cellulolysis (*Ruminococcus, Butyrivibrio*, and *Orpinomyces*), proteolysis (*Prevotella* and Entodiniinae), lactate production (*Streptococcus* and *Selenomonas*), as well as methylotrophic archaea (Methanomassiliicoccaceae). This microbial adaptation indicated that pasture degradation is a complex process which requires a diverse consortium of microbes working together. The correlations between the abundance of microbial taxa and rumen fermentation parameters were not consistent across diets suggesting a metabolic plasticity which allowed microbes to adapt to different substrates and to shift their fermentation products. The core microbiota was composed of 34, 9, and 13 genera for bacteria, methanogens, and fungi, respectively, which were shared by all sheep, independent of diet. This systems biology approach adds a new dimension to our understanding of the rumen microbial interactions and may provide new clues to describe the mode of action of future nutritional interventions.

## Introduction

Rumen bacteria, archaea, anaerobic fungi, protozoa and phages make up the complex microbial ecosystem which enables ruminants to efficiently utilize forage. This multi-kingdom rumen microbiota has been described as “the most elegant and highly evolved cellulose-digesting system in nature” (Weimer et al., 2009). As a result ruminants are among the few livestock types which potentially do not compete for human edible foods (Gill et al., 2010). Fresh grass has traditionally been a major feedstuff for ruminants and grazing systems generally have a positive perception in society in terms of animal well-being (Somers et al., 2005). However, in the context of growing demand for animal products, modern ruminant production systems based on large scale farms tend to replace fresh pastures with preserved forages, such as hay, supplemented with concentrate feeds during certain periods of the year when the grass is unavailable (e.g., winter time) or when a greater control of the diet is required (e.g., lactation period). However, the decision “to graze or not to graze” is often arbitrary without taking into consideration the impact on the rumen microbiota, feed efficiency and on the environment (Pol-van Dasselaar et al., 2008).

Several studies have described the rumen microbial changes over the transition from forage to concentrate diets (Fernando et al., 2010; Zhu et al., 2017) however much less research has been conducted to explore the microbial adaptation to fresh forage diets. Differences between fresh grass and grass hay in terms of rumen fermentation and digestion of nutrients have been extensively reported (Minson, 1990; Givens et al., 2000). A recent *in vitro* study linked the kinetics of feed colonization with changes in the rumen microbiota and we demonstrated that a fresh grass diet, in comparison to grass hay, can accelerate the microbial feed colonization of ingested feed (Belanche et al., 2017) leading to higher feed digestibility, microbial protein synthesis and lower methane emissions (Belanche et al., 2016b). It is well-known that different diets provide different primary substrates for fermentation *in vitro*; however animal physiological features, such as feeding behavior, rumen temperature, pH or feed retention time may cause different inter-relationships between the rumen microbial groups *in vivo* to those observed *in vitro*. Moreover, due to the complexity of the rumen ecosystem, most studies focus on highlighting properties of individual microbial species in response to dietary treatments, leaving the interactions within and between the microbial communities unexplored. Thus, there is a need to implement new methodological approaches to reveal the impact of nutritional strategies on the whole rumen microbiota under farm conditions.

Recent studies across different habitats have demonstrated that a better picture of the whole function of a microbial ecosystem can be achieved by combined interpretation of quantitative (taxon abundance and diversity) and qualitative approaches, such as core microbiota and network/co-occurrence analysis. It is likely that similar to human gut (Turnbaugh et al., 2009), there is a “rumen core microbiota” that remains stable regardless of difference in diet or host genetics (Petri et al., 2013; Henderson et al., 2015). However, only few studies have implemented this concept in ruminants using a limited number of experimental animals (Taxis et al., 2015; Tapio et al., 2017a). Microbial networks have successfully been used to predict the dynamics and structure of oceanic plankton ecosystems (Lima-Mendez et al., 2015), soil environments (Barberán et al., 2012) and to identify taxa interactions within the human gut in health and disease (Baldassano and Bassett, 2016). Preliminary multi-kingdom studies of the co-occurrence patterns of bacteria, archaea and eukaryotic rumen microbes across different ruminant species (Kittelmann et al., 2013) and diets (Kumar et al., 2015) have revealed that the rumen microbial community is shaped by various biotic and abiotic factors which still need to be better understood (Henderson et al., 2015).

The aim of this study was to implement multi-kingdom community analyses (including bacteria, methanogens, fungi and protozoa) in order to investigate the rumen microbial adaptation when animals face a feeding challenge consisting of a shift from a conventional non-grazing diet to a grazing situation. Moreover, the use of a relatively large number of experimental animals allowed identification of the core rumen microbiota and generation of robust microbial networks based on co-occurrence patterns between microbial taxa.

## Materials and Methods

### Animals and Diets

Animal procedures were conducted in accordance with the Home Office Scientific Procedures, Act 1986 and were authorized by the Aberystwyth University Ethics Committee (PLL 40/3653; PIL 40/9798). Twenty four Aberdale ewes of an average body weight of 68 ± 6.7 kg were used in a straight through experimental design consisting of two 45-days periods. During the first period all sheep received a diet composed of ryegrass hay (*Lolium perenne*) offered *ad libitum* and supplemented with 725 g DM of commercial concentrate per animal and day (Ewemaster Gold 19 Nuts, Wynnstay, Aberystwyth, UK). Concentrate was divided into two equal meals fed at 09:00 and 17:00 h, respectively. During the second period all sheep grazed a perennial ryegrass pasture. Ryegrass hay was obtained from a second cut of a ryegrass monoculture (*Lolium perenne* L. cv. AberMagic, Germinal GB Ltd, Lincoln, UK) located in Aberystwyth, UK (52°43′N, 4°02′W) and had a target maturity of reproductive stage R1-index 3.1 which shows a visible spikelet of inflorescence emergence (Moore and Moser, 1995). After cutting, ryegrass was left to dry on the field for 7 days, tedded and packed into 20 kg bales. The grazing period took place on the same ryegrass pasture from which the hay was harvested and both forages had similar maturity stage. All animals remained together in the same flock and had free access to fresh water. Feed chemical composition was determined (Belanche et al., 2013) and reported in Table 1.

**Table 1:** Feed composition (in % of DM).

**Feed**	**Concentrate**	**Ryegrass hay**	**Ryegrass pasture**
Dry matter (% FM)	91.4	86.9	17.8
Organic matter	92.1	93.6	90.4
Crude protein	18.9	6.1	11.4
Water soluble carbohydrates	6.7	13.6	17.1
Starch	22.1	–	–
Carbon	44.3	44.2	44.6
Carbon/Nitrogen ratio	14.6	45.4	9.3
Neutral detergent fiber	51.5	64.4	51.0
Acid detergent fiber	14.6	34.6	22.1

### Rumen Sampling and Analyses

At the end of each period, rumen fluid (200 ml per sheep) was withdrawn by orogastric intubation prior to morning feeding (09:00 h). Rumen fluid was filtered through cheesecloth, pH was measured and five subsamples were taken: the first subsample (40 ml) was immediately snap-frozen in liquid N for DNA extraction and microbial characterization. The second (4 ml) was diluted with 1 ml deproteinizing solution (20% orthophosphoric acid containing 10 mM of 2-ethylbutyric acid) for volatile fatty acid (VFA) determination. The third subsample (1 ml) was diluted with 0.6 ml of trichloroacetate (25% w:vol) for ammonia analysis. The fourth subsample (1 ml) was snap-frozen for lactate determination, and the last subsample (1 ml) was diluted with 1 ml of formalin (9.25% and NaCl 0.9% w:vol) for protozoal optical counting and classification (Dehority, 1993).

Rumen concentrations of protozoa, ammonia, VFA and lactate were determined as previously described (Belanche et al., 2013). Rumen DNA was extracted from freeze-dried samples (Yu and Morrison, 2004) and quantitative PCR was used (Table S1). To determine the absolute concentration of bacteria, methanogens, anaerobic fungi, and protozoa (Belanche et al., 2015).

### Next Generation Sequencing (NGS)

Rumen bacteria, methanogenic archaea, and fungal communities were analyzed using NGS DNA metabarcoding as previously described (Belanche et al., 2016a; Detheridge et al., 2016). Briefly, for bacteria and methanogens sequencing of the V1-V2 and the V2-V3 hypervariable regions of the 16S rRNA were performed respectively, while for the fungi the D1 variable region of the large subunit (28S LSU) of the rRNA locus was amplified (Table S1). Amplicons were pooled in equimolar concentrations, purified using an E-gel and analyzed and quantified using a Bioanalyser 2100 (Agilent Technologies, Santa Clara, USA). Library preparation and sequencing was performed using an Ion Torrent system and 2 PGM Sequencing 316™ v2chips were used for bacteria and fungi, respectively (Life Technologies Ltd, Paisley, UK), while one smaller 314™ v2 chip was used for methanogens. Sequences were trimmed at 300 bp length (200 bp for fungi) and Mothur software was used the quality filtering consisting of: maximum 10 homo-polymers, quality Q15 average over 30 bp window and no mismatches with the primer/barcoding were allowed (Schloss et al., 2009). Error rate was controlled using UParse (error = 1). Chimera checking was performed using Uchime and sequences were clustered into OTUs at 97% identity using Uclust (Edgar et al., 2011). Taxonomic classification was conducted using the Ribosomal Database Project II classifier containing curated sequences of the bacterial 16S and fungal LSU sequences (Cole et al., 2013), while the RIM-DB database was used for methanogens (Seedorf et al., 2014). Singletons were removed and only taxonomical annotations with a confidence (bootstrap value) above 80% were considered, otherwise were considered as unclassified. This approach allowed methanogens to be mostly classified at the species level, while bacteria and fungi were mostly classified at the genus level. To maximize the comparability across samples, the number of reads per sample was manually normalized to the sample with the lowest number of reads. Raw sequences reads were deposited at EBI Short Read Archive (PRJEB27535).

### Calculations and Statistical Analyses

Protozoal cell counts, quantitative PCR data and the number of reads of each microbial taxon were log-transformed to assume normality. Rumen fermentation and microbial data were analyzed based on a repeated-measures ANOVA using the MIXED models of Genstat 18th Edition (VSN International, Hemel Hempstead, UK) as follows:

$$\begin{array}{l} Y_{ijk}=\mu +D_{i}+A_{j}+e_{ij} \end{array}$$

where *Y*_*ijk*_is the dependent, continuous variable (*n* = 24), μ is the overall mean, *D*_*i*_ is the fixed effect of the diet (*i* = CON vs. PAS), *A*_*l*_ is the random effect of the animal (*j* = 1–24) and *e*_*ijkl*_ is the residual error. Significant effects were declared at *P* < 0.05 and tendency to difference at *P* < 0.1.

Sequence data were log-transformed, and the diet and animal effects were studied: to determine the impact of the diet on the microbial community structure a non-parametric permutational multivariate analysis of variance (PERMANOVA) was conducted based on the Bray-Curtis dissimilarity (PRIMER-6 Ltd., Plymouth, UK). Statistical significance was calculated after 999 random permutations of residuals under the reduced model using the Monte Carlo test. A heatmap containing the taxonomical information and the community structure (R statistics, Vegan package) was performed for graphical interpretation. A canonical correspondence analysis (CCA) was also conducted to explore the relationships between the structure of microbial community and the fermentation pattern. The significance of each variable was also calculated after 999 random permutations (R statistics; Vegan package). For microbial taxa abundance, data were log-transformed and False Discovery Rate was minimized using the Bonferroni statistical test. Spearman correlation coefficients were calculated to assess the relationships between the ruminal abundance of the main microbial taxa and the fermentation data.

In order to decipher the structure of the rumen microbial community and the multi-kingdom interactions, network analyses were performed under different dietary situations (Belanche et al., 2017). This approach, based on the co-occurrence of bacterial, methanogen, fungal and protozoal taxa described inter-relationships based on their positive and negative correlations. For each microbial network, only those taxa (mainly at the genus level) present in more than 50% of the individuals were included. Spearman correlation analysis was performed between all microbial taxa using log-transformed data and only correlation coefficients larger than 0.5 and adjusted *P*-values below 0.05 were further included in the correlation network. Network analysis was generated using R package igraph (Csardi and Nepusz, 2006). Microbial network complexity was described in terms of number of nodes (genera), number of edges (positive or negative correlations), betweenness (measure of centrality in a graph based on shortest paths) and contribution to the total community. The core microbiota was calculated as those genera (or species) present in >95% animals (Turnbaugh et al., 2007).

## Results

### Rumen Fermentation and Microbial Diversity

Diet modified most rumen fermentation parameters (Table 1): the CON diet promoted a high fermentation rate in terms of total VFA as well as high lactate, H_2_ production and molar proportion of acetate (*P* < 0.001). On the contrary, the shift to the PAS diet promoted a higher rumen ammonia concentration and higher molar proportions of propionate, butyrate, branched-chained VFA (iso-butyrate and iso-valerate) as well as higher ratio of D to L lactate (*P* < 0.001).

Quantitative PCR (Table 2) showed a higher ruminal concentration of bacteria (*P* <0.001) and protozoa (*P* =0.011) in sheep fed with the PAS than with the CON diet. On the contrary the CON diet promoted a higher concentration of anaerobic fungi per mg of DM (*P* = 0.027). The absolute concentration of methanogens was not affected by the diet, but their relative abundance with respect to bacteria was higher for the PAS diet (*P*<0.001). Next generation sequencing produced 2.46, 0.37, and 1.64 million high quality sequences and samples were normalized at 12,500, 1,098, and 8,901 reads per sample for bacteria, methanogens, and fungi, respectively. After this normalization, Good's coverage index remained high indicating that sequencing depth was sufficient and comparable across samples. Sheep fed on the PAS diet, in comparison to the CON diet, showed higher bacterial and fungal diversity indexes (*P* < 0.05). Similarly, PAS diet also promoted higher methanogen richness (*P* < 0.001) but with a lower Evenness than in the CON diet (*P* = 0.008) suggesting the co-occurrence of highly abundant methanogen species together with very low abundant species.

**Table 2:** Effects of the diet on the rumen fermentation, absolute abundance, and alpha diversity of the main microbial groups in sheep.

**Item**	**CON**	**PAS**	**SED**	***P-value***
Body weight (kg)	65.7	70.1	1.038	<0.001
**RUMEN FERMENTATION**				
pH	6.86	6.77	0.052	0.117
Ammonia-N (mg/l)	26.8	105	6.285	<0.001
VFA (mM)	87.7	59.4	3.284	<0.001
**Molar proportion (%)**				
Acetate	71.4	59.4	0.963	<0.001
Propionate	15.4	20.1	1.053	<0.001
Butyrate	9.39	13.5	0.240	<0.001
Iso-butyrate	1.70	2.23	0.106	<0.001
Valerate	0.69	1.36	0.035	<0.001
Iso-valerate	1.14	2.15	0.080	<0.001
Caproate	0.25	0.75	0.096	<0.001
Iso-caproate	0.01	0.44	0.027	<0.001
Lactate (mM)	11.9	5.91	1.561	<0.001
D/L Lactate ratio	0.30	2.25	0.036	<0.001
H_2_ production*^a^* (mM)	173	117	6.200	<0.001
**CONCENTRATIONS (LOG COPIES/MG DM)**				
Bacteria	8.38	8.91	0.104	<0.001
Methanogens	5.90	6.28	0.702	0.593
Methanogens (10^3^ × ΔC^T^)	0.31	1.00	0.130	<0.001
Anaerobic fungi	6.90	5.85	0.446	0.027
Protozoa	5.10	8.49	1.226	0.011
**BACTERIAL ALPHA DIVERSITY**				
Richness	1940	2161	73.77	0.006
Shannon	6.10	6.35	0.080	0.006
Evenness	0.81	0.83	0.008	0.015
Simpson	0.99	0.99	0.001	0.077
Good's	0.93	0.92	0.004	0.040
**METHANOGENS ALPHA DIVERSITY**				
Richness	25.4	28.0	0.668	<0.001
Shannon	2.33	2.27	0.054	0.263
Evenness	0.72	0.68	0.014	0.008
Simpson	0.84	0.83	0.012	0.307
Good's	0.86	0.79	0.020	0.002
**FUNGAL ALPHA DIVERSITY**				
Richness	66.7	87.6	6.906	0.006
Shannon	1.37	1.97	0.079	<0.001
Evenness	0.33	0.44	0.016	<0.001
Simpson	0.61	0.73	0.025	<0.001
Good's	0.75	0.76	0.043	0.794

*CON, ryegrass hay diet supplemented with concentrate; PAS, ryegrass pasture*.

a *Hydrogen production stoichiometrically calculated (Marty and Demeyer, 1973)*.

### Core Microbiota and Microbial Network

**Figure 1:**
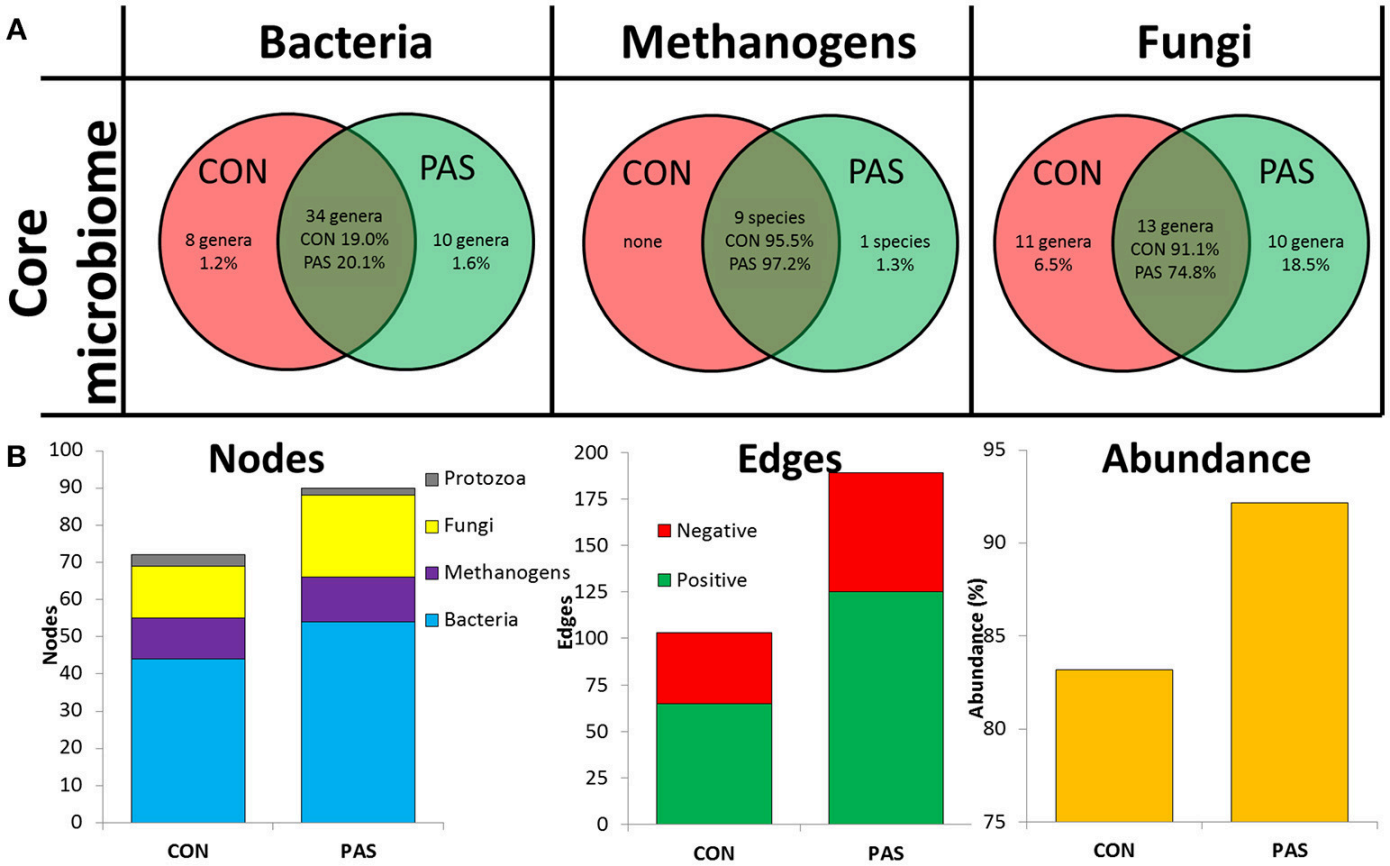
**(A)** Venn diagrams describing the effect of the diet on the core microbial communities in the rumen. **(B)** Microbial network data describing the effect of the diet on the number of nodes, edges and microbial abundance in the rumen of sheep. Networks were generated based on those genera which positively or negatively correlated (*r* > 0.5 and adjusted-*P* < 0.05). CON, ryegrass hay diet supplemented with concentrate; PAS, ryegrass pasture.

The core microbiota present across the vast majority of the individuals was affected by the diet, but to different magnitudes according to the microbial community considered (Figure 1A). The core bacterial microbiota was composed of 34 genera across diets and represented ~20% of the bacterial community. Moreover, eight (*Microbacterium, Olsenella, Alkalitalea, Porphyromonas, Elusimicrobium, Mogibacterium, Robinsoniella*, and *Leptotrichia*) and 10 additional genera (*Actinomyces, Barnesiella, Alloprevotella, Sphingobacterium, Howardella, Hydrogenoanaerobacterium, Mitsuokella, Lawsonia, Campylobacter*, and *Endomicrobium*), representing 1.2 and 1.6% of the community respectively, formed the diet-specific core bacterial community for CON and PAS diets, respectively. The core methanogen community was composed of nine species which represented nearly the entire methanogen population (96%) across diets and there was not a diet-specific core community. The core fungal community was composed of 13 genera and was the most affected by the diet because this community represented 91.1% of the fungal community in the CON diet but only 74.8% in the PAS diet. Moreover, a substantial diet-specific core community was observed comprising 11 and 10 fungal genera and representing 6.5 and 18.5% for the CON and the PAS diets, respectively.

Microbial network analysis showed that sheep fed a CON diet, in comparison to a PAS diet, had a lower microbial network complexity in terms of nodes, edges, average number of neighbors and in the abundance of the rumen microbiota taking part of this network (Figure 1B). Most of the nodes belonged to bacterial genera (61%) followed by fungi (22%), methanogens (14%) and protozoa (3%) (Table S3 and Figure S4). These figures were modulated by the diet with an increase in the number of fungal nodes and positive edges with the PAS diet.

### Bacterial Community Structure and Taxonomy

**Figure 2:**
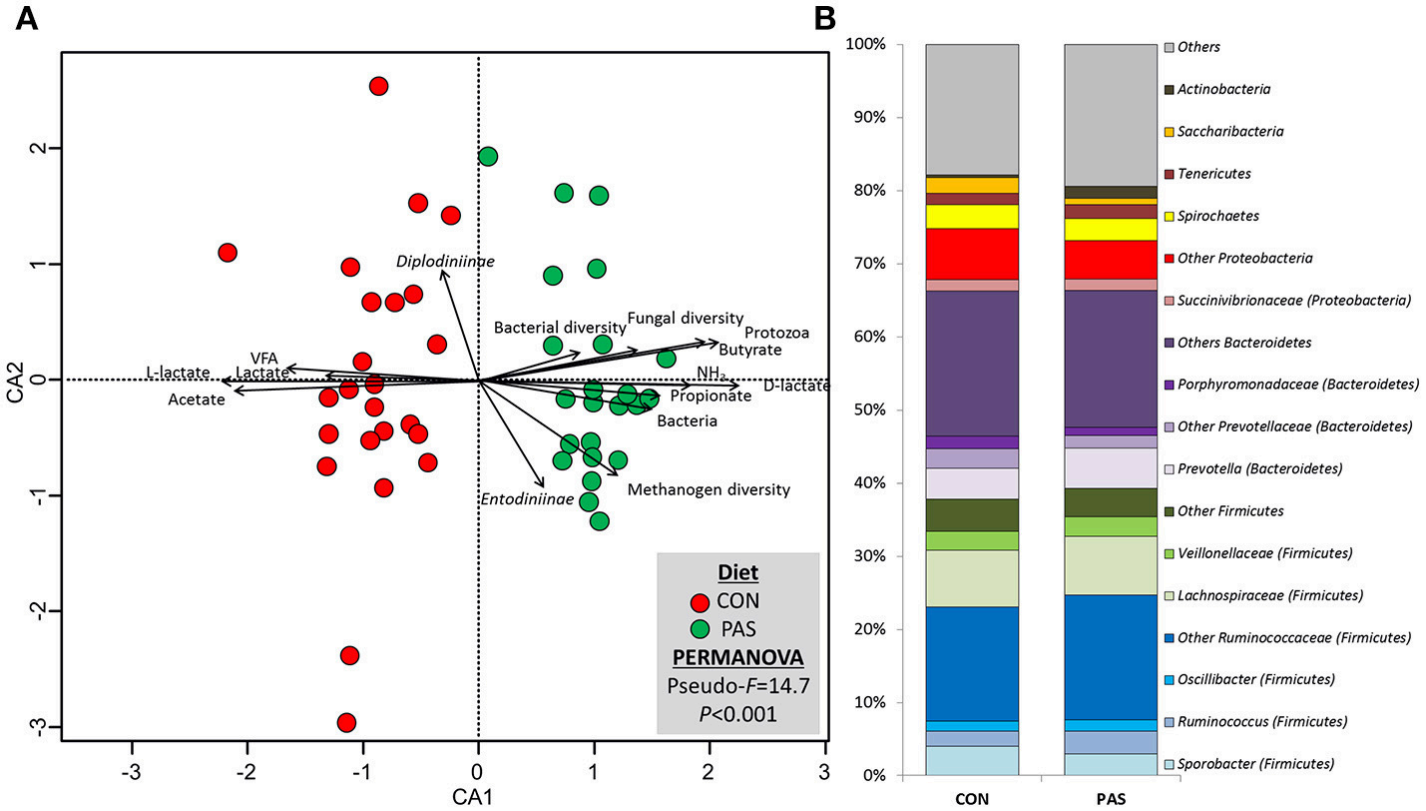
**(A)** Canonical correspondence analysis illustrating the effect of the diet on the relationship between the structure of the bacterial community and the rumen fermentation. PERMANOVA indicating the effect of the diet based on the Bray-Curtis dissimilarity. **(B)** Effect of the diet on the abundance of the main bacterial taxa in sheep. CON, ryegrass hay supplemented with concentrate; PAS, ryegrass pasture.

PERMANOVA revealed substantial differences in the community structure between animals for the bacterial community (*P* = 0.028), but also for the methanogen (*P* = 0.001) and fungal communities (*P* = 0.020). This animal effect was considered as random and is not further discussed. The bacterial community structure was also visibly affected by the diet (Figure 2; Table S2) to a greater extent than observed for methanogen and fungal communities, as noted by the lower Pseudo-*F* values (14.7 vs. 32.8 vs. 33.9) and similarity values (36.6 vs. 70.7 vs. 45.3) for bacteria, methanogens, and fungi, respectively. As a result, the CCA (Figure 2A) and the heatmap (Figure S1) of the bacterial community based on the dissimilarly showed a clear separation between samples from animals fed the CON and the PAS diet. CCA analysis showed that the structure of the bacterial community in sheep fed the CON diet was positively correlated with the rumen concentration of VFA, total lactate, L-lactate and acetate molar proportion, while for the PAS diet the bacterial structure was positively correlated with the rumen concentration of ammonia, D-lactate, propionate and butyrate molar proportion as well as to the abundance of bacteria and protozoa and the bacterial and fungal diversity (richness).

With regard to the abundance of bacterial taxa (Table 3 and Figure 2B), diet modified the concentration of 55% of the taxa which were present in abundances above 0.05%. The shift from a CON to a PAS diet did not promote a change in the proportions of Firmicutes and Bacteroidetes but led to a greater abundance of Actinobacteria (mainly *Actinomyces*), certain Firmicutes families (Ruminococaceae, Erysipelotrichaceae) and genera (e.g., *Succiniclasticum, Solobacterium, Butyrivibrio, Lachnobacterium, Syntrochococcus, Pseudoflavonifractor, Ruminococcus*, or *Selenomonas*) as well as some Bacteroidetes (*Phocaeicola* and *Paraprevotella*) and Proteobacteria genera (*Ruminobacter*). On the contrary, CON diets promoted higher levels of the phyla Saccharibacteria and Fusobacteria, as well as several genera (e.g., *Anaerotronchus, Butyricicoccus, Ethanoligenenes, Sporobacter*, or *Succinivibrio*). Only the abundance of few genera showed a consistent and similar correlation with fermentation parameters under both dietary situations. Examples of this consistency across diets were the positive correlation of the lactate-producer *Selenomonas* with the rumen lactate and butyrate levels; or the negative correlation between *Prevotella* and the BCVFA concentration. However, most microbes showed correlations with rumen fermentation parameters under only one dietary treatment.

**Table 3:** Effects of the diet on the relative abundance of the main bacterial taxa and their correlation with fermentation data in the rumen of sheep.

			**ANOVA**				***Correlations***															
			**Diet**				**pH**		**NH**_****3****_		**VFA**		**Acetate**		**Propionate**		**Butyrate**		**BCVFA**		**Lactate**	
**Phylum**	**Family**	**Genus**	**CON**	**PAS**	**SED**	***P*-value**	**CON**	**PAS**	**CON**	**PAS**	**CON**	**PAS**	**CON**	**PAS**	**CON**	**PAS**	**CON**	**PAS**	**CON**	**PAS**	**CON**	**PAS**
Actinobacteria			1.40	2.08	0.100	<0.001																−0.50
	Actinomycetaceae	*Actinomyces*	0.55	2.03	0.148	<0.001																−0.46
	Coriobacteriaceae		0.83	0.76	0.094	0.490				0.38		0.34								0.37		
		*Enterorhabdus*	0.21	0.39	0.067	0.014				0.44										0.63		
		*Olsenella*	0.74	0.53	0.098	0.049						0.37		−0.34					−0.37			
	Microbacteriaceae	*Microbacterium*	0.89	0.47	0.105	<0.001					0.36				0.38		−0.31				−0.39	
Bacteroidetes			3.42	3.41	0.022	0.630		0.32		−0.34								0.44				0.32
	Bacteroidaceae	*Anaerorhabdus*	0.53	0.45	0.116	0.521			−0.34						0.53				−0.34			
	Bacteroidales	*Phocaeicola*	1.06	1.47	0.061	<0.001																
	Flavobacteriaceae		0.52	0.33	0.102	0.070								0.48		−0.47			0.31			
		*Empedobacter*	0.20	0.22	0.070	0.783				0.38			0.32				−0.32	−0.38		0.35		
	Marinilabiliaceae		1.11	0.88	0.136	0.106			0.34													
		*Alkalitalea*	0.83	0.74	0.163	0.602			0.46		0.34		−0.56	0.39	0.57							−0.34
	Porphyromonadaceae		2.17	2.02	0.047	0.005																
		*Barnesiella*	0.53	0.94	0.132	0.004																
		*Porphyromonas*	1.19	0.09	0.102	<0.001					−0.49		0.44		−0.41				0.43			
	Prevotellaceae		2.79	2.82	0.039	0.477				−0.58			0.42				−0.36	0.34		−0.31	−0.34	
		*Alloprevotella*	1.12	0.57	0.175	0.004	0.35		0.33		−0.38					0.38			0.45		0.36	
		*Hallella*	0.18	0.09	0.083	0.260																
		*Paraprevotella*	0.72	1.20	0.097	<0.001			0.40										0.39			0.38
		*Prevotella*	2.58	2.68	0.053	0.054				−0.54	0.32							0.32	−0.33	−0.35	−0.33	
	Sphingobacteriaceae		0.92	0.88	0.103	0.704			0.33													
		*Sphingobacterium*	0.39	0.50	0.087	0.222																
Saccharibacteria			2.22	1.89	0.071	<0.001																
Elusimicrobia			0.97	0.94	0.091	0.743					0.37											
	Endomicrobium		0.48	0.76	0.099	0.009								0.32								−0.31
	Elusimicrobiaceae	*Elusimicrobium*	0.77	0.51	0.104	0.022			0.48										0.36			
Fibrobacteres	Fibrobacteraceae	*Fibrobacter*	1.10	1.08	0.141	0.995							−0.43	0.52		−0.56	0.32			0.32		
Firmicutes			3.81	3.81	0.014	0.721						−0.35										
	Acidaminococcaceae		1.38	1.40	0.055	0.710														0.42		0.40
		*Succiniclasticum*	1.17	1.40	0.051	<0.001					0.31		0.31				−0.34			0.49	−0.38	0.45
		*Succinispira*	0.75	0.00	0.109	<0.001																
	Clostridiales XII	*Guggenheimella*	0.87	0.61	0.069	0.001								0.43		−0.62				0.34		
	Clostridiales XIII		1.29	1.37	0.061	0.249			−0.35	−0.35								0.57		0.54		0.49
		*Anaerovorax*	0.69	0.87	0.064	0.011										−0.32						
		*Mogibacterium*	1.01	1.04	0.121	0.833												0.59				0.50
	Erysipelotrichaceae		1.66	1.97	0.087	0.002		0.39				−0.49		0.47		−0.44						
		*Erysipelotrichaceae*	1.46	1.67	0.137	0.144																
		*Solobacterium*	0.88	1.37	0.086	<0.001		0.32						0.51	0.53	−0.42			−0.38	0.33		
	Lachnospiraceae		2.85	2.87	0.034	0.479	−0.57		−0.31		0.57					−0.34						
		*Anaerostipes*	0.22	0.60	0.070	<0.001											−0.48				−0.36	
		*Butyrivibrio*	0.25	0.48	0.083	0.010																
		*Cellulosilyticum*	0.13	0.22	0.057	0.109								0.40		−0.40						
		*Clostridium XlVa*	0.37	0.09	0.074	0.001					−0.48		0.40									
		*Howardella*	0.55	0.59	0.049	0.421				0.33	0.41				−0.34			−0.56				−0.58
		*Lachnobacterium*	1.07	1.25	0.064	0.010																
		*Lachnospiracea*	1.70	2.00	0.070	<0.001																−0.39
		*Lactonifactor*	1.13	1.11	0.079	0.786		0.34				−0.44		0.48		−0.53	0.40			0.44	0.49	
		*Pseudobutyrivibrio*	0.86	0.60	0.090	0.009																
		*Robinsoniella*	1.27	0.07	0.151	<0.001	−0.63				0.52											
		*Roseburia*	1.45	1.56	0.087	0.229			−0.41													
		*Syntrophococcus*	0.85	1.31	0.057	<0.001	−0.35				0.58				0.32					−0.34		
	Ruminococcaceae		3.33	3.37	0.016	0.038	0.31		0.55			−0.39		0.32								−0.31
		*Acetanaerobacterium*	1.15	1.01	0.091	0.144				0.55										0.51		
		*Anaerotruncus*	1.67	1.35	0.092	0.002	0.44				−0.32											
		*Butyricicoccus*	1.09	0.74	0.076	<0.001			0.47							−0.42			0.50	0.34		
		*Clostridium IV*	0.84	0.72	0.078	0.14		0.33				−0.34		0.49		−0.63				0.32		
		*Ethanoligenens*	1.25	0.95	0.073	<0.001	−0.42							0.64		−0.41		−0.47				−0.38
		*Flavonifractor*	1.93	2.03	0.049	0.066		−0.57			0.39	0.38								−0.38		
		*Oscillibacter*	2.05	2.15	0.056	0.067				0.36												
		*Papillibacter*	0.22	0.34	0.094	0.228						−0.36		0.40		−0.34						
		*Pseudoflavonifractor*	1.25	1.39	0.064	0.043						−0.37				−0.46				0.33		
		*Ruminococcus*	2.28	2.44	0.036	<0.001	0.45			−0.32			0.42				−0.57				−0.49	
		*Sporobacter*	2.54	2.43	0.044	0.018																
		*Streptococcus*	0.08	0.23	0.064	0.021						0.33		−0.33								
	Veillonellaceae		2.34	2.40	0.051	0.221																
		*Anaerovibrio*	0.25	0.23	0.062	0.826				0.43									−0.31	0.45		
		*Mitsuokella*	0.10	0.96	0.055	<0.001												−0.39				
		*Selenomonas*	0.63	1.05	0.075	<0.001						0.33		−0.59		0.50	0.50	0.48			0.54	0.43
Fusobacteria	Leptotrichiaceae	*Leptotrichia*	1.10	0.23	0.065	<0.001			0.33						−0.34					0.40		
Proteobacteria			2.88	2.80	0.038	0.032				0.43								−0.33				
	Campylobacteraceae	*Campylobacter*	0.30	0.41	0.025	<0.001	−0.38		−0.34					−0.37		0.41	0.32		−0.47			
	Desulfovibrionaceae		0.63	0.68	0.063	0.453		0.36				−0.41	0.32			−0.47						0.42
		*Lawsonia*	0.60	0.59	0.070	0.868		0.33				−0.39	0.34					−0.36				
	Moraxellaceae	*Acinetobacter*	0.16	0.46	0.070	<0.001												0.34				
	Pasteurellaceae		0.81	0.44	0.106	0.002			0.36					0.39		−0.46				0.53		
		*Bibersteinia*	0.72	0.31	0.109	<0.001			0.32	0.33				0.33		−0.44				0.59		
		*Mannheimia*	0.20	0.11	0.050	0.083																
	Sphingomonadaceae	*Sphingomonas*	2.08	1.85	0.077	0.008			0.44	0.40								−0.37	0.64	0.34		
	Succinivibrionaceae		2.13	2.10	0.087	0.681				0.46												
		*Ruminobacter*	1.77	2.03	0.103	0.024				0.39	−0.32			−0.31								
		*Succinimonas*	0.86	0.52	0.092	0.001							0.43				−0.31				−0.32	
		*Succinivibrio*	1.49	0.73	0.122	<0.001								0.36		−0.43						
Spirochaetes			2.47	2.45	0.032	0.673			0.34				−0.65	0.36	0.57							−0.38
	Spirochaetaceae		2.34	2.39	0.029	0.074	−0.45				0.47		−0.32		0.36							
		*Sphaerochaeta*	1.20	1.22	0.051	0.714				0.59	0.46											
		*Treponema*	2.09	2.19	0.041	0.017		0.43		−0.38	0.33											
SR1			0.49	0.33	0.107	0.188							0.35	0.40		−0.53						
Synergistetes			0.33	0.40	0.075	0.427	0.36								−0.39				0.34			
Tenericutes	Anaeroplasmataceae	*Anaeroplasma*	2.13	2.20	0.062	0.253		0.42		−0.36									−0.38			

*The number of reads per sample was normalized to 12,500 and log-transformed. Minor taxa are not shown (< 0.05%). Only Spearman correlations with r > 0.30 and P < 0.05 are shown (n = 24). CON, ryegrass hay diet supplemented with concentrate; PAS, ryegrass pasture*.

*Green and red values indicate positive and negative correlations, respectively*.

### Methanogens and Protozoa Community Structure and Taxonomy

**Figure 3:**
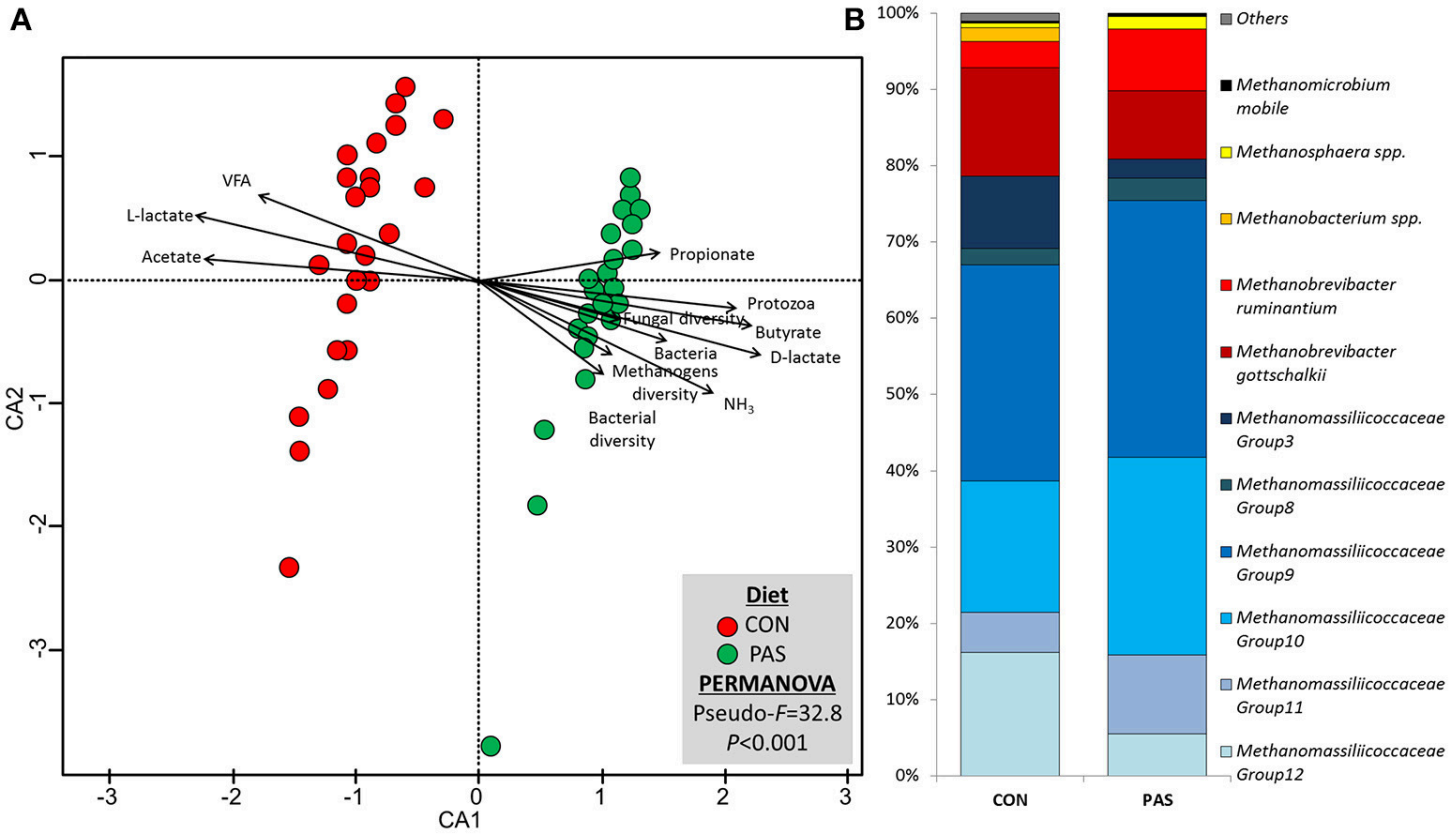
**(A)** Canonical correspondence analysis illustrating the effect of the diet on the relationship between the structure of the methanogen community and the rumen fermentation. PERMANOVA indicating the effect of the diet based on the Bray-Curtis dissimilarity. **(B)** Effect of the diet on the abundance of the main methanogen taxa in sheep. CON, ryegrass hay supplemented with concentrate; PAS, ryegrass pasture.

Diet promoted a significant change in the structure of the methanogen community (Table S2; Figure S2). CCA showed a positive correlation between the structure of the methanogens community in sheep fed the CON diet (based on the Bray-Curtis dissimilarity) and the rumen concentration of total VFA, L-lactate and acetate (Figure 3A). On the other hand, the methanogen community structure in sheep fed a PAS diet (in terms of dissimilarity) positively correlated with the ruminal concentration of ammonia, D-lactate, bacteria and protozoa, the butyrate and propionate molar proportion and the bacterial, methanogens and fungal diversities. Similar to bacteria, the abundances of 55% of the methanogen taxa were significantly affected by the diet (Table 4). In particular (Figure 3B), the shift from CON to PAS diet promoted a decrease in the abundance of *Methanobacterium, Methanobrevibacter gottschalkii*, Methanobassiliicocccaceae Group 12 and *Methanomicrobium mobile*, along with a decrease in *Methanobrevibacter bovis koreani, Methanobrevibacter ruminantium, Methanosphaera*, and several Methanomassiliicoccaceae species (Groups 9, 10, and 11). The correlation analysis showed that when animals were fed the CON diet most Methanobacteriaceae taxa positively correlated with propionate and negatively with lactate. Two Methanomassiliicoccaceae taxa (Groups 3 and 8) positively correlated with acetate and negatively with propionate, while the opposite was true for the Groups 9 and 11, however these observations only occurred when sheep were fed a PAS but not with a CON diet.

**Table 4:** Effect of the diet on the relative abundance of the main methanogen and protozoal taxa and their correlation with fermentation data in the rumen of sheep.

			**ANOVA**				***Correlations***															
			**Diet**				**pH**		**Ammonia**		**VFA**		**Acetate**		**Propionate**		**Butyrate**		**BCVFA**		**Lactate**	
**Family**	**Genus**	**Species**	**CON**	**PAS**	**SED**	***P*****-value**	**CON**	**PAS**	**CON**	**PAS**	**CON**	**PAS**	**CON**	**PAS**	**CON**	**PAS**	**CON**	**PAS**	**CON**	**PAS**	**CON**	**PAS**
**METHANOGENS**																						
Methanobacteriaceae			2.24	2.24	0.068	0.977									0.32						−0.59	
		*Methanobacterium*	0.74	0.01	0.139	<0.001																
	*Methanobrevibacter*		2.19	2.19	0.070	0.99									0.37						−0.48	
		*M. boviskoreani*	0.00	0.58	0.074	<0.001																
		*M. gottschalkii*	2.09	1.92	0.066	0.015				−0.35					0.45						−0.35	
		*M. ruminantium*	1.37	1.78	0.099	<0.001												−0.31			−0.54	
		*M. wolinii*	0.22	0.10	0.087	0.153														−0.45		
	*Methanosphaera*		0.71	1.20	0.085	<0.001									0.42			−0.41			−0.60	
		*Group5*	0.16	0.40	0.058	<0.001								−0.34	0.56	0.37						
		*ISO3F5*	0.67	1.14	0.088	<0.001											−0.41			−0.63		
Methanomassiliicoccaceae			2.88	2.94	0.034	0.074			−0.39									0.42				0.59
	*Group 3a*		1.90	1.29	0.083	<0.001					−0.47			0.34		−0.37						
	*Group 8*		1.10	1.27	0.140	0.217			−0.33	0.39	0.32			0.35		−0.46						
	*Group 9*		2.35	2.51	0.052	0.008								−0.34		0.32			−0.36			
	*Group 10*		2.13	2.42	0.099	0.010										−0.39						
	*Group 11*		1.56	2.01	0.082	<0.001				−0.35				−0.34		0.37		0.45	−0.33			
	*Group 11*	*M. alvus*	0.00	0.99	0.092	<0.001				−0.46								0.32				
	*Group 11*	*BRNA1*	0.03	0.00	0.018	0.171																
	*Group 11*	*CRM1*	0.40	0.43	0.148	0.773																
	*Group 11*	*ISO4G11*	1.46	1.92	0.083	<0.001				−0.34					0.39			0.37	−0.37			
	*Group 12*	*ISO4H5*	2.05	1.55	0.135	0.001					−0.35										−0.43	
Methanocorpusculaceae		*Methanocorpusculum*	0.03	0.00	0.018	0.171													0.34			
Methanomicrobiaceae	*Methanomicrobium*	*M. mobile*	0.47	0.00	0.119	<0.001	−0.31						0.39		−0.37		0.40		−0.31	0.30		
Methanosarcinaceae	*Methanimicrococcus*	*M. blatticola*	0.08	0.04	0.044	0.382													−0.53			
**PROTOZOA**																						
Protozoa (log cells/ml)			5.32	5.93	0.046	<0.001				−0.39				0.39			0.39					0.39
Subf. Entodiniinae (%)			89.6	90.8	1.511	0.469				0.40				−0.39	0.46	0.38						
Subf. Diplodiniinae (%)			2.65	1.87	0.787	0.260																
*Isotricha* (%)			0.50	0.69	0.249	0.448			−0.32										−0.33	−0.33		
*Dasytricha* (%)			7.21	6.65	1.078	0.599								0.47	−0.51	−0.45						

*The number of reads per sample was normalized to 1,098 and log-transformed. Only Spearman correlations with r > 0.30 and P < 0.05 are shown (n = 24). CON, ryegrass hay diet supplemented with concentrate; PAS, ryegrass pasture*.

*Green and red values indicate positive and negative correlations, respectively*.

All sheep showed an abundant and highly diverse protozoal population (Table 4). Optical protozoal counting showed higher protozoal levels with the PAS than with the CON diet. This protozoal concentration positively correlated with the butyrate and lactate concentrations but only when sheep were fed the CON diet. A positively correlation was noted between propionate and Entodiniinae, while this propionate negatively correlated with *Dasytricha* spp. across diets.

### Fungal Community Structure and Taxonomy

**Figure 4:**
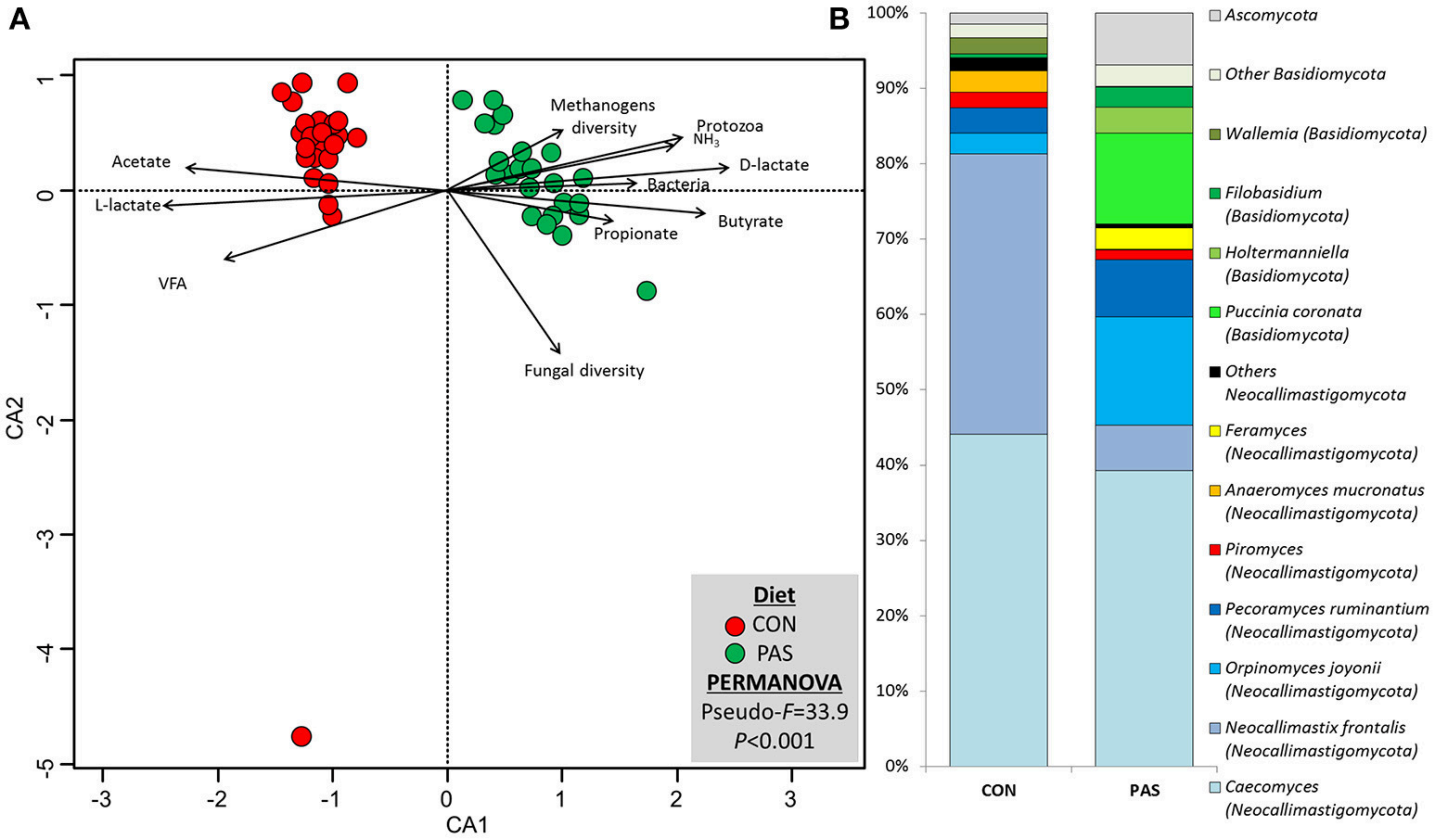
**(A)** Canonical correspondence analysis illustrating the effect of the diet on the relationship between the structure of the fungal community and the rumen fermentation. PERMANOVA indicating the effect of the diet based on the Bray-Curtis dissimilarity. **(B)** Effect of the diet on the abundance of the main fungal taxa in sheep. CON, ryegrass hay supplemented with concentrate; PAS, ryegrass pasture.

The change from a CON to a PAS diet also promoted a shift in the fungal community structure (Figure S3; Table S2). CCA showed that the fungal community structure in the rumen of sheep fed the CON diet (based on the Bray-Curtis dissimilarity) was positively correlated with the fermentation rate (total VFA, acetate and L-lactate), while in sheep fed PAS this community was positively correlated with the rumen concentration of bacteria, protozoa, ammonia, D-lactate, propionate and butyrate and methanogen diversity (Figure 4A). The concentration of most fungal taxa (91%) was affected by the diet (Table 5 and Figure 4B): sheep fed a CON diet, in comparison to a PAS diet, had higher levels of anaerobic fungi (Neocallimastigomycota), notably *Neocallimastix, Piromyces, Anaeromyces*, and *Buwchfawromyces*. Conversely, Ascomycota, Basidiomycota, and some Neocallimastigomycota (*Orpinomyces, Pecoramyces*, and *Feramyces*) were more abundant in PAS-fed sheep. Ruminal concentration of total anaerobic fungi was positively correlated with acetate and BCVFA and negatively correlated with propionate molar proportion with both diets. On the contrary, yeast was positively correlated with propionate and negatively with acetate and BCVFA molar proportions across diets.

**Table 5:** Effect of the diet on the relative abundance of the main fungal taxa and their correlation with fermentation data in the rumen of sheep.

			**ANOVA**				***Correlations***															
			**Diet**				**pH**		**Ammonia**		**VFA**		**Acetate**		**Propionate**		**Butyrate**		**BCVFA**		**Lactate**	
**Phylum**	**Family**	**Genus**	**CON**	**PAS**	**SED**	***P-*value**	**CON**	**PAS**	**CON**	**PAS**	**CON**	**PAS**	**CON**	**PAS**	**CON**	**PAS**	**CON**	**PAS**	**CON**	**PAS**	**CON**	**PAS**
Ascomycota			1.93	2.66	0.084	<0.001			−0.31				−0.42	−0.48	0.38	0.45			−0.54	−0.43		
	Davidiellaceae		0.59	1.56	0.100	<0.001			−0.43				−0.32			0.33			−0.37	−0.65		
		*Cladosporium*	0.25	1.42	0.094	<0.001	−0.47		−0.47		0.39					0.37				−0.62		
		*Davidiella*	0.5	1.02	0.094	<0.001			−0.34	−0.32			−0.43		0.39				−0.35	−0.66		
	Mycosphaerellaceae		0.56	1.71	0.109	<0.001	−0.36				0.32				0.49				−0.61	−0.36		
		*Cymadothea*	0.01	1.62	0.110	<0.001														−0.37		
	Didymellaceae	*Didymella*	0.68	1.93	0.083	<0.001							−0.33	−0.50		0.53			−0.41	−0.37		
	Phaeosphaeriaceae	*Phaeosphaeria*	1.11	2.04	0.071	<0.001			−0.47			0.47		−0.54	0.35	0.54			−0.70	−0.31		
	Pleosporaceae		0.81	1.74	0.100	<0.001	−0.36						−0.34	−0.36	0.33				−0.39			
		*Alternaria*	0.66	1.15	0.113	<0.001	−0.43			−0.41			−0.40		0.31			0.43	−0.36	−0.44		0.35
		*Neoascochyta*	0.25	1.03	0.106	<0.001				0.32		0.53		−0.32								
		*Pyrenochaeta*	0.33	1.27	0.086	<0.001					0.37		−0.35	−0.48		0.46						
	Helotiaceae		1.08	1.41	0.104	0.005	−0.39				0.35		−0.39	−0.39	0.48	0.40		0.32	−0.51	−0.51		
		*Articulospora*	0.5	1.16	0.102	<0.001							−0.41	−0.41	0.38	0.45			−0.46	−0.47		
		*Cadophora*	0.98	1.04	0.096	0.558	−0.44			−0.47	0.39		−0.36	−0.47	0.48	0.41		0.44	−0.45	−0.51		0.37
	Saccharomycetaceae		1.18	0.92	0.140	0.089			−0.46									−0.33	−0.42			
		*Debaryomyces*	1.01	0.21	0.119	<0.001			−0.45										−0.37			
	Trichocomaceae	0.85	0.66	0.144	0.226	−0.46						−0.42	−0.65		0.54	0.31	0.34	−0.49	−0.47		
Basidiomycota			2.45	3.19	0.093	<0.001	−0.34						−0.48	−0.33	0.42	0.43	0.36		−0.36	−0.43		
	Tricholomataceae		0.48	0.75	0.118	0.037				−0.43			0.34		−0.68			0.31	0.44	−0.37		0.40
		*Squamanita*	0.34	0.58	0.099	0.029				−0.48	−0.40				−0.58		0.35	0.31			0.50	
	Cystobasidiaceae	*Cystobasidium*	0.68	1.14	0.094	<0.001	−0.45		−0.42		0.32		−0.47	−0.38	0.40	0.34	0.43	0.38	−0.47	−0.31		0.35
	Leucosporidiales	*Leucosporidium*	0.93	1.27	0.081	<0.001	−0.48		−0.42			0.33	−0.44	−0.63	0.56	0.56		0.32	−0.56	−0.42		
	Sporidiobolales	*Rhodotorula*	0.16	0.79	0.100	<0.001				−0.31				−0.34		0.33		0.34		−0.53		
		*Sporobolomyces*	1.48	1.13	0.094	0.001	−0.36						−0.52	−0.32	0.60	0.41	0.40		−0.42	−0.41		
	Pucciniaceae	*Pucciniacoronata*	0.44	2.87	0.114	<0.001					−0.46				−0.39					−0.54	0.35	
	Cystofilobasidiaceae	*Cystofilobasidium*	0.69	1.84	0.079	<0.001	−0.43			0.34		0.48	−0.39	−0.57	0.47	0.56	0.34					
	Mrakiaceae		1.37	0.56	0.093	<0.001	−0.45			−0.31			−0.57		0.56		0.37	0.59				0.40
		*Itersonilia*	1.08	0.27	0.086	<0.001	−0.49						−0.57		0.56		0.42					
		*Mrakia*	1.07	0.33	0.095	<0.001	−0.46			−0.40		−0.38	−0.49		0.57		0.36			−0.53		
	Filobasidiaceae	*Filobasidium*	1.48	2.28	0.083	<0.001	−0.45				0.32	0.38	−0.53	−0.56	0.54	0.69	0.36		−0.55	−0.51		
	Holtermanniales	*Holtermanniella*	0.62	2.37	0.083	<0.001	−0.49		−0.39			0.35	−0.50	−0.44	0.34	0.59	0.54			−0.41	0.42	
	Bulleribasidiaceae	*Dioszegia*	1.19	1.24	0.109	0.643	−0.45				0.31		−0.42	−0.43	0.46	0.36		0.33	−0.33	−0.38		
	Wallemiaceae	*Wallemia*	1.94	0.66	0.126	<0.001																
Neocallimastigomycota	Neocallimastigaceae		3.92	3.79	0.021	<0.001			0.37				0.47	0.40	−0.44	−0.48	−0.33		0.51	0.65		
		*Anaeromyces mucronatus*	2.19	0.33	0.149	<0.001																
		*Buwchfawromyces*	1.69	0.86	0.134	<0.001								0.42		−0.47						
		*Caecomyces*	3.57	3.49	0.053	0.119		0.49														
		*Feramyces*	0.44	1.16	0.234	0.005	−0.39	−0.42			0.42	0.44				0.38						
		*Neocallimastix frontalis*	3.48	2.56	0.105	<0.001						−0.33		0.48		−0.34						
		*Orpinomyces joyonii*	1.63	2.89	0.154	<0.001	−0.45				0.46			0.31		−0.45						
		*Pecoramyces ruminantium*	1.61	2.51	0.203	<0.001	−0.43							0.32		−0.37						
		*Piromyces*	2.21	1.93	0.106	0.015	−0.34	0.45		−0.31		−0.41										
**CLASSIFICATION BY ECOLOGY**																						
Anaerobic fungi			3.92	3.79	0.021	<0.001			0.37				0.47	0.40	−0.44	−0.48	−0.33		0.51	0.65		
Pathogens			1.49	2.98	0.107	<0.001	−0.38			−0.31			−0.49		0.46		0.34		−0.37	−0.57		
Saprophytes			2.23	2.41	0.100	0.074						0.33	−0.41	−0.59	0.31	0.61			−0.34	−0.49		
Yeast			1.62	1.95	0.085	<0.001			−0.37			0.31	−0.47	−0.54	0.43	0.59	0.39		−0.49			

*The number of reads per sample was normalized to 8,901 and log-transformed. Minor taxa are not shown (< 0.05%). Only Spearman correlations with r > 0.30 and P < 0.05 are shown (n = 24). CON, ryegrass hay diet supplemented with concentrate; PAS, ryegrass pasture*.

*Green and red values indicate positive and negative correlations, respectively*.

## Discussion

### Rumen Core Microbiota

This study explored the concept of “core rumen microbiota” similar to that hypothesized for the human gut (Turnbaugh et al., 2009). A core bacterial community was identified in the rumen of sheep across diets. This community was formed by 35 dominant genera including *Prevotella, Sporobacter, Ruminococcus, Anaeroplasma, Treponema, Ruminobacter, Succinivibrio, Fibrobacter, Selenomonas*, representing 20% of the bacterial community. These core community members are mostly similar to those identified in a previous study in which 32 genera were shared across 16 dairy cows fed different diets (Jami and Mizrahi, 2012). Moreover, most of these genera have also been found in other culture-dependent studies (Tajima et al., 2001; Pitta et al., 2010). More interestingly, our study found that diet-specific core communities were small in size (<2%) indicating that most of the adaptation processes to the PAS diet did not rely on the replacement of bacterial genera but on increasing the bacterial concentration and diversity or on modifying the taxa abundance and/or their activity.

Methanogenic archaea are the only organisms able to produce methane (Hook et al., 2010). Our study identified a simple methanogen community in terms of diversity (26.7 OTUs), moreover the core community was also limited in diversity (only nine species comprising *M. gottschalkii, M. ruminantium, Methanosphaera ISO3F5*, and Methanomassiliicoccaceae groups 3a, 8, 9, 11, and 12), but large in size (95.5% of the total methanogen abundance). As a result, no diet-specific core community was observed. These findings agree with previous observations (Tapio et al., 2017a) and indicated the presence of a highly conserved core methanogen community. However, the diet consumed by the ruminant affected both the overall structure of the methanogens community and the abundance of 55% of the methanogens taxa indicating a larger diet adaptation process than reported for the bacterial community (Popova et al., 2013).

Fungi represent 10–20% of the rumen microbiota (Rezaeian et al., 2004), we found that the great majority of the fungal species (81.9%) belonged to anaerobic fungi (Neocallimastigomycota) including most of the genera described so far in ruminants (Edwards et al., 2017). Most of these anaerobic fungi formed the core fungal community composed of 13 genera which were shared across all sheep, representing over three quarters of the total fungal abundance. However, this study also detected a smaller proportion of fungi that were likely to have been ingested with the feed materials, including plant-pathogens (5.5%), saprotrophs (2.4%), yeast (0.7%) and other species of unclassified fungi (9.5%). One example of a plant-pathogenic fungus was the crown rust *Puccinia coronata* (Roderick and Thomas, 1997) which was more abundant in PAS than in CON sheep, while the xerophilic basidiomycetous yeast *Wallemia* was more abundant in CON sheep because is commonly found in dried feeds (Zajc and Gunde-Cimerman, 2018). The use of generic fungal primers has been validated for rumen studies (Edwards et al., 2017) because although most fungal species entering the rumen with the feed are obligate aerobes, and thus considered to be transient and non-functional (Bauchop, 1979), some of them (e.g., yeast) can have modulatory effects on the rumen function (Newbold et al., 1996).

### Rumen Microbiota in Animals fed Non-grazing Diets

This section describes the rumen microbiota when animals were fed conventional diets based on grass hay plus concentrate in order to further evaluate the adaptation process when animals were shifted to a grazing diet, which is considered the main objective of this study. Under our experimental conditions, ryegrass hay had lower protein (−47%) and soluble carbohydrates (−21%) and higher neutral (+26%) and acid-detergent concentrations (+56%) than fresh ryegrass. This nutrient loss is mainly due to degradation of sugars due to plant respiration, oxidation of fatty acids and loss of leaves during raking (Pizarro and James, 1972). As a result of this, under farm conditions hay is often supplemented with concentrate, as conducted in this study. This concentrate supplementation in the CON diet led to high rumen VFA and lactate concentrations due to the supply of high levels of rumen fermentable material (mainly as starch). This observation was supported by the higher proliferation of starch-degrading bacteria, such as Saccharibacteria, *Succinovibrio*, and *Succinomonas* in CON than in PAS fed sheep, as well as profound differences in the rumen microbial community structure and fermentation.

CCA revealed that structure of the bacterial community in the CON diet was positively associated with a higher VFA concentration and negatively correlated with ammonia and bacterial richness. According to Shabat et al. (2016) this situation should indicate a more efficient rumen function based on the study of the rumen microbiota of 146 milking cows. However, they also noted that efficient cows had a higher propionate/acetate ratio, aspect that was not observed in our CON-fed animals. Firkins et al. (2007) reported that starch supplementation in the rumen favors the ammonia incorporation by the rumen bacteria (Firkins et al., 2007) and could explain the low ammonia-N concentration observed in sheep fed CON diet (26.8 g N/l). In particular, Oba and Allen (2003) indicated that increasing the percentage of grain in the ration increased microbial N flow to the duodenum by about 30%. Recycling of blood urea N back to the rumen can partially ameliorate this low ammonia-N availability in the rumen, but increases the likelihood that amino-N precursors might become more limiting for some microbes and ultimately limit the microbial protein synthesis in ruminants feed high amounts of rumen-degradable starch (Firkins et al., 2007). Rumen protozoa are unable to use ammonia as N source (Williams and Coleman, 1992), although anaerobic fungi and bacteria can uptake ammonia N, presence of amino-N can substantially increase their growth rate (Dijkerman et al., 1996; Atasoglu et al., 1999). Thus, the low concentrations of rumen protozoa and bacteria, along with the low fungal diversity observed in CON-fed sheep may indicate that amino-N could represent the limiting factor for microbial growth. Our study identified various microbes which could suffer this limitation (*Alloprevotella, Butyricicoccus, Fusobacteria, Bibersteinia, Sphingomonas, or Spirochaetes*) because these bacterial genera are not involved in proteolysis but showed a positive correlated with the rumen ammonia concentration in the CON-fed animals (Griswold et al., 2003). These findings agree with a previous study (Belanche et al., 2012c) in which a protein shortage (from 110 to 80% of the N requirements) decreased not only the ruminal concentration of prokaryotes (bacteria and methanogens) but also the concentration and diversity of eukaryotes (protozoa and anaerobic fungi) which led to a decreased feed digestibility in dairy cows.

Methanogenic archaea stabilize the fermentation process in the rumen by utilizing H_2_ and reducing H_2_ partial pressure. The stoichiometrical calculations based on the VFA concentration suggested that CON-fed sheep had a high availability H_2_ in the rumen for archaeal utilization (Marty and Demeyer, 1973). As a result, higher abundances of *M. gottschalkii, Methanobacterium*, and *Methanomicrobium* were noted when sheep were fed a CON rather than PAS diet. All three species are hydrogenotrophic using CO_2_ as the electron acceptor and H_2_ produced by protozoa, bacteria and fungi as the electron donor (Liu and Whitman, 2008). Most hydrogenotrophic methanogens can also utilize formate as electron donor (equivalent to H_2_ +CO_2_) by the activity of formate dehydrogenase (Liu and Whitman, 2008). In our study *Methanomicrobium* was negatively correlated with propionate, which together whit the low propionate concentration in the rumen suggested that this product was unlikely to be a substantial H_2_ sink in the CON diet. It was described that *M. gottschalkii* clade is associated with high methane emissions to a greater extent than the overall methanogens concentration (Tapio et al., 2017b). However, other microbiological features, such as low rumen levels of Proteobacteria and high levels of H_2_ producing bacteria and certain rumen protozoa and anaerobic fungi genera have also been associated with high methane emissions (Tapio et al., 2017b), aspect that was not noted when sheep were fed the CON diet. These findings suggest that rumen microbiota analysis can help to understand the rumen methanogenesis, but not yet in a predictive manner.

Anaerobic fungi are among the most potent fiber degrading organisms known to date due to their extensive set of enzymes for the degradation of plant structural polymers (Solomon et al., 2016) Moreover anaerobic fungi possess amylolytic (Gordon and Phillips, 1998) and proteolytic activity (Gruninger et al., 2014) which make this community dependent of the nutrient supply as noted in our study. The high concentration of anaerobic fungi in the CON-fed sheep was mostly due to increased levels of *Anaeromyces, Neocallimastix, Buwchfawromyces*, and *Piromyces*. While the substrate preference of the newly described genus *Buwchfawromyces* is still unknown (Callaghan et al., 2015), *Anaeromyces* and *Piromyces* have a preference for glucose and fructose (Solomon et al., 2016). On the contrary *Neocallimastix* is a monocentric fungus, able utilize a wider spectrum of substrates (Edwards et al., 2017), such as cellulose, xylose, glucose, starch, grass and straw. Despite their different substrate preferences, all these fungal genera have been associated with increased production of formate, acetate and lactate (Edwards et al., 2017). This fungal activity could partially explain the unexpected higher acetate levels observed in CON than in PAS fed animals as a positive correlation between the fungal community structure and acetate was noted in CON-fed sheep.

The correlation analysis also showed a general lack of agreement between the abundance of bacterial, methanogens, fungal and protozoal taxa and the rumen fermentation parameters across diets suggesting a shift in their metabolic pathways driven by the diet. This observation suggests that functional diversity may occur even with similar taxonomical distribution and thus expands a previous hypothesis which stated that taxonomic differences mask functional similarity (Taxis et al., 2015). Therefore, the notion that the availability of rumen degradable energy and protein for microbial protein synthesis which is commonly used in most feeding systems ignores how microbial populations change with varying nutrient supply (i.e., starch, ammonia-N, peptides) or how these populations can change their metabolism under different conditions (Firkins et al., 2007). Our findings suggest that these microbial adaptations should be revisited if new feeding systems are developed in the future.

### Microbial Adaptation to Grazing Diets

Fresh ryegrass represents a less fibrous and higher quality forage than ryegrass hay in terms of protein and soluble carbohydrates contents. The higher rumen protein availability in the PAS diet led to increased levels of protein degradation products, such as isobutyrate (+31%), isovalerate (+88%) and ammonia (+3.9-fold) which seems to indicate a greater protein breakdown than in the CON diet. Fresh forages are capable of degrading part of its own protein within the first 2 h of ruminal incubation, irrespective of the microbial colonization due to the presence of active plant enzymes (Kingston-Smith et al., 2003). This study revealed that several microbes seem to be associated with these proteolytic processes since CCA and correlation analysis showed that the structure of the bacterial, methanogens and fungal communities, as well as the protozoal family Entodiniinae were positively correlated with the ammonia concentration (Hobson and Stewart, 2012). Moreover, the PAS diet also led to greater rumen molar concentration of butyrate (+44%) which could indirectly alter the proteolysis and deamination rates because presence of butyrate-producing bacteria (mainly *Bacteroidetes* in this study) have been described as modulators of the populations of hyperammonia-producing bacteria (Firkins et al., 2007).

Regarding rumen energy metabolism, the lack of concentrate supplementation in grazing animals led to lower rumen VFA (−32%) and lactate concentration (−50%) likely due to a lower starch supply. Both lactate isomers (D and L) are produced in the rumen but in presence of soluble sugar most D-lactate, and some L-lactate, is metabolized into propionate as the main product (Counotte et al., 1983). Thus, the lower lactate concentration, together with the high levels of lactate producers, such as *Streptococcus* and lactate utilizers, such as *Selenomonas* in the PAS diet may suggests that most of the lactate was transformed into propionate (+31%) in grazing animals, possibly due to the high availability of soluble carbohydrates (Huws et al., 2009). Several studies have demonstrated a decrease in the Firmicutes/Bacteroidetes ratio and in the bacterial diversity during the transition from high forage to high grain diets (Fernando et al., 2010; Tapio et al., 2017a). In our study the proportions of these two phyla remained constant indicating that starch supply in the CON diet may be partially compensated in the PAS diet by the higher water soluble carbohydrates content and feed digestibility reported for fresh ryegrass in comparison to ryegrass hay (Belanche et al., 2016b).

The shift from non-grazing to grazing diets caused an increase in the bacterial concentration and diversity (+221 OTUs) which has been suggested as an adaptation strategy to digest forage diets (Belanche et al., 2012b). In this dietary situation the bacterial community structure was positively correlated to parameters associated with high microbial complexity, such as the protozoal concentration or the bacterial, fungal and methanogens diversities. A similar correlation pattern was observed for the methanogen and fungal communities suggesting that rumen microbial adaptation to degradation of fresh pasture implies a larger number of microbes working together than for non-grazing diets (McAllister et al., 1994). The current study supported the concept that amino acids availability in the rumen stimulates fibrolytic bacteria, especially those involved in the degradation of hemicelluloses (Griswold et al., 2003). In particular, the adaptation to the PAS diet led to increased levels of cellulolytic (*Ruminococcus* and *Butyrivibrio*) and proteolytic bacteria (*Prevotella*), but also amylolytic (*Ruminobacter, Succiniclasticum*) and lactate producers (*Streptococcus* and *Selenomonas*). Microbial network analysis also showed a higher overall complexity with PAS rather than CON diets (+18 nodes and +86 edges) mostly due to the presence of more bacterial nodes (+10). This finding disagrees with a recent *in vitro* study in which a similar bacterial network complexity was observed in rumen liquid incubated with fresh grass or grass hay (Belanche et al., 2017). This discrepancy may be due to the different forage to concentrate ratio used in the two studies.

Regarding methanogen community, the transition from the CON to the PAS diet increased the methanogen relative abundance and diversity (+2.6 OTUs). Wallace et al. (2014) suggested that methane emissions are positively correlated (*R* = 0.49) with the Archaea:Bacteria ratio. According to this observation the PAS diet should promote as much as 2.2-folds higher methane emissions than the CON diet. However, a recent publication reported that the prediction of rumen methanogenesis is a more complex task which requires the study of the genes involved in the hydrogenotrophic methane synthesis along with the methylotrophic methanogens and VFA profile to better predict methane emissions across a range of dietary conditions (Auffret et al., 2018). Our study revealed that the adaptation process to the grazing diet was coupled with increases in the abundance of *M. ruminantium, M. bovis, Methanosphaera* and Methanossiliicoccaceae Groups 9 and 11. Although *Methanobrevibacter* is hydrogenotrophic, *Methanosphaera*, and most members of the Methanomassiliicoccaceae family are methylotrophic, reducing methylamines and methanol with H_2_ (Paul et al., 2015). In the rumen, methylamines are derived from glycine betaine and choline, which are present in plant membranes, while methanol is a product of pectin hydrolysis by protozoa and the esterase activity of bacteria (Poulsen et al., 2013). These substrates are highly abundant in fresh forages and may have favored the proliferation of these species in animals fed the PAS diet. Moreover, the methanogens community structure (dissimilarity) in animals fed PAS diet was positively correlated with propionate molar proportion suggesting that may act as an alternative H_2_ sink. Therefore, the increase in the propionate molar proportion along with a decrease in the VFA concentration when animals shifted from CON to PAS diet led to lower H_2_ production (−32%) which could partially reduce the expected differences in methane emissions based on the methanogen concentrations (Wallace et al., 2014; Auffret et al., 2018).

Microbial adaptation to the PAS diet also led to an increase in protozoal cell concentration in which the Entoniniinae family represented 90.8% of this community. This protozoal population is actively involved in protein (Belanche et al., 2012a) and carbohydrate degradation (Ushida et al., 1987), as supported by the positive correlation with the ammonia and propionate levels observed in our study. It has been estimated that between 9 and 25% of rumen methanogens are associated with protozoa (Newbold et al., 1995) and produce ~37% of the methane emissions (Finlay et al., 1994). In previous publications we demonstrated that the presence of rumen protozoa can increase the methanogen concentration and diversity (Belanche et al., 2012b, 2014), as noted in the present study, and suggests a higher interspecies H_2_ transfer in animals fed the PAS diet (Morgavi et al., 2010).

The early stages of feed colonization process is challenge to rumen microbes, particularly when fresh and little damaged plant material is ingested by the ruminant (Edwards et al., 2008). Anaerobic fungi can facilitate this colonization process because their rhizoids have the ability to physically penetrate plant structural barriers (Callaghan et al., 2015). Thus, fungi promote other microbes in further plant colonization (Belanche et al., 2017). Our study agreed with these observations and revealed that the rumen fungal adaptation from the CON to the PAS diet was a multi-factorial process consisting of an increase in diversity (+20.9 OTUs) and a shift in the community structure. This new fungal community positively correlated with other microbial groups, such as protozoa, bacteria, and methanogens highlighting the aforementioned symbiotic relations. Cheng et al. (2009) demonstrated that the activity of anaerobic fungi was enhanced by methanogenic archaea which are known to physically attach to fungal biomass (Li et al., 2017). A highly complex fungal community has also been shown to improve feed digestibility, feed efficiency, dairy weight gain, and milk production (Puniya et al., 2015). In a previous study using Automated Ribosomal Intergenic Spacer Analysis we observed an increase in the fungal diversity when a starch-rich diet was replaced by a fiber-rich diet in dairy cattle (Belanche et al., 2012c). However, the use of NGS in the present study allowed identifying the anaerobic fungi favored by the PAS diet, such as *Orpinomyces, Pecoramyces*, and *Feramyces*. This increase in *Orpinomyces* may relate to its long life cycle and more indeterminate (polycentric) mode of growth which favors its proliferation in animals grazing fresh forage, due to the longer rumen retention time (Dey et al., 2004). *Orpinomyces* has been shown to be able to utilize various substrates, forming H_2_, CO_2_, formate, acetate, lactate, and ethanol as fermentation products (Edwards et al., 2017) as noted in the our study. It is also possible that predation of fungal zoospores by rumen protozoa (Lee et al., 2001) may lead to shift toward genera, such as *Orpinomyces* with lower reliance on sporulation due to their indeterminate growth, at the expense of monocentric taxa, such as *Neocallimastix* with more limited capacity for development of extensive thalli.

Microbial network analysis revealed that the adaptation to the PAS diet promoted an increase in the importance of the fungal community (+8 fungal nodes) which highly interacted with multiple bacterial and methanogens taxa leading to an increase in the overall network complexity and the proportion of positive symbiotic interactions (+5%). Kumar et al. (2015) also noted an increase in the co-occurrence between anaerobic fungi, methanogens and bacterial genera when dairy cows were fed a high forage diet rather than a concentrate diet. However, this later study also indicated that co-occurrence studies can also be affected by biotic factors, such as the age of the animal or the number of lactations suggesting that the rumen microbiota is a dynamic ecosystem which is still far from been fully understood.

## Implications

Here we show that there are various layers of microbial community analysis currently available, such as taxa abundance, diversity, core community, network, and correlation analyses which can provide a wealth of information about the rumen community adaptation to different dietary situations. In conventional non-grazing systems supplemented with starch-rich concentrate the rumen microbiota had low complexity in terms of diversity and network density suggesting a more efficient nutrient utilization by the rumen microbes. This scenario was associated with higher VFA production and lactate fermentation. However, when animals shifted to a grazing diet the rumen microbiota experienced an adaptation process in order to face the challenge of colonizing and digest fresh plant material. This process consisted on an increase in the microbial concentration (higher abundance of bacteria, methanogens and protozoa), diversity and network complexity. This adaptation originated from a shift in the rumen microbiota promoted a modification in the multi-kingdom microbial interactions and fermentative activities (higher proteolysis) which could have relevant productive implications; however functional genomics would be required to definitively link these observations with the feed degradation pathways.

## Author Contributions

AB and CN designed the experiment. AB conducted the research and wrote the manuscript. AB, AK-S, GG, and CN reviewed the manuscript. AB had primary responsibility for the final content. All authors read and approved the final manuscript.

### Conflict of Interest Statement

The authors declare that the research was conducted in the absence of any commercial or financial relationships that could be construed as a potential conflict of interest.
