# Supplementary material for: A Multi-Kingdom Study Reveals the Plasticity of the Rumen Microbiota in Response to a Shift From Non-grazing to Grazing Diets in Sheep
Source: Front Microbiol. 2019 Feb 11;10:122. doi: 10.3389/fmicb.2019.00122 (PMC6396721; doi:10.3389/fmicb.2019.00122)
Supplement: Supplementary file 1 [file Table_1.DOCX]

**ONLINE SUPPORTING MATERIAL**

**Table S1.** Primers used for quantitative PCR and Ion-Torrent Next Generation Sequencing.

| **Target** | **Author** | **Forward Primer** | **Reverse Primer** | **T^a^** | | **Amplicon (bp)** |
| --- | --- | --- | --- | --- | --- | --- |
| **Quantitative PCR** |  |  |  | |  |  |
| Total bacteria | ([Maeda et al., 2003](#_ENREF_4)) | GTGSTGCAYGGYTGTCGTCA | ACGTCRTCCMCACCTTCCTC | | 61 | 150 |
| Methanogens | ([Denman et al., 2007](#_ENREF_2)) | TTCGGTGGATCDCARAGRGC | GBARGTCGWAWCCGTAGAATCC | | 56 | 140 |
| Anaerobic fungi | ([Denman and McSweeney, 2006](#_ENREF_1)) | GAGGAAGTAAAAGTCGTAACAAGGTTTC | CAAATTCACAAAGGGTAGGATGATT | | 62 | 120 |
| Protozoa | ([Sylvester et al., 2004](#_ENREF_6)) | GCTTTCGWTGGTAGTGTATT | CTTGCCCTCYAATCGTWCT | | 55 | 223 |
| **Ion Torrent NGS** |  |  |  | |  |  |
| Bacterial primers | ([Spear et al., 2008](#_ENREF_5)) | AGAGTTTGATCMTGGCTCAG | CTGCTGCCTYCCGTA | | 58 | 348 |
| Bacterial Adaptors |  | CCATCTCATCCCTGCGTGTCTCCGACTCAG | CCTCTCTATGGGCAGTCGGTGAT | |  |  |
| Methanogens primers | ([Wright and Pimm, 2003](#_ENREF_7)) | GCTCAGTAACACGTGG | GWATTACCGCGGCKGCTG | | 58 | 433 |
| Methanogens adaptors |  | CCATCTCATCCCTGCGTGTCTCCGACTCAG | CCTCTCTATGGGCAGTCGGTGAT | |  |  |
| Fungal primers | ([Detheridge et al., 2016](#_ENREF_3)) | CYYAGTARCTGCGAGTGAAG | GAGCTG- CATTCCCAAACAA | | 52 | 200-220 |
| Fungal adaptors |  | CCATCTCATCCCTGCGTGTCTCCGAC | CCTCTCTATGGGCAGTCGGTGAT | |  |  |
|  |  |  |  | |  |  |

**Table S2.** PERMANOVA describing the effect of the diet on the structure of the rumen microbial communities

|  | **Similarity** |  |  | **Significance** |  |
| --- | --- | --- | --- | --- | --- |
| **Community** | **CON vs CON** | **CON vs PAS** | **PAS vs PAS** | **Pseudo-F** | **P-value** |
| Bacteria | 45.0 | 36.3 | 53.2 | 14.7 | 0.001 |
| Methanogens | 79.5 | 70.7 | 80.2 | 32.8 | 0.001 |
| Fungi | 65.4 | 46.3 | 63.3 | 33.9 | 0.001 |

CON: Ryegrasss hay diet supplemented with concentrate; PAS; ryegrass pasture.

**Table S3.** Summary of the microbial network parameters illustrating the effect of the diet on the relationships between the different microbial groups in the rumen of sheep.

| **Item** | **CON** | **PAS** |
| --- | --- | --- |
| Total nodes | 72 | 90 |
| Bacteria | 44 | 54 |
| Methanogens | 11 | 12 |
| Fungi | 14 | 22 |
| Protozoa | 3 | 2 |
| Proportions (%) |  |  |
| Bacteria | 61.1 | 60.0 |
| Methanogens | 15.3 | 13.3 |
| Fungi | 19.4 | 24.4 |
| Protozoa | 4.17 | 2.22 |
| Total abundance (%) | 83.2 | 92.1 |
| Total edges | 103 | 189 |
| Positive | 65 | 125 |
| Negative | 38 | 64 |
| Proportions (%) |  |  |
| Positive | 63.1 | 66.1 |
| Negative | 36.9 | 33.9 |
| Other network indicators |  |  |
| Number of neighbours | 3.561 | 5.739 |
| Density | 0.040 | 0.047 |
| Diameter | 10.00 | 8.000 |
| Transitivity | 0.256 | 0.317 |
| Modularity | 0.664 | 0.534 |
| Assortativity | 0.281 | 0.224 |
| Central degree | 0.058 | 0.133 |
| Central closeness | 0.051 | 0.058 |
| Central betweenness | 0.277 | 0.178 |
| Central eigen | 0.817 | 0.864 |

CON, ryegrass hay diet supplemented with concentrate; PAS, ryegrass pasture


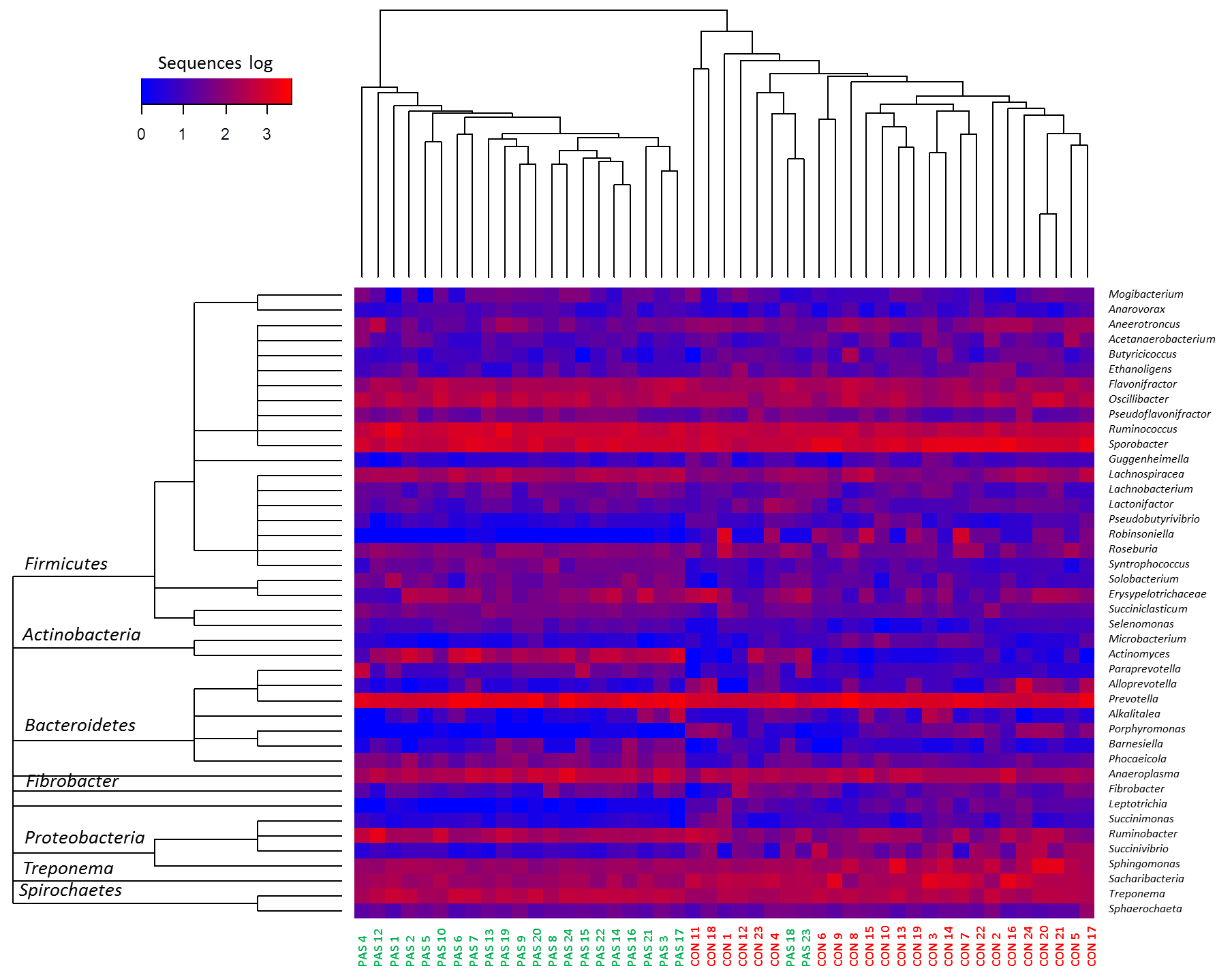


**Figure S1.** Heatmap describing the effect of the diet on the structure of the bacterial community in the rumen of sheep. CON: ryegrass hay diet supplemented with concentrate; PAS; ryegrass pasture. Dendrogram based on the UPGMA clustering of the Bray-Curtis distances. The number of reads per sample was normalized, log transformed and minor genera discarded (<0.05%).


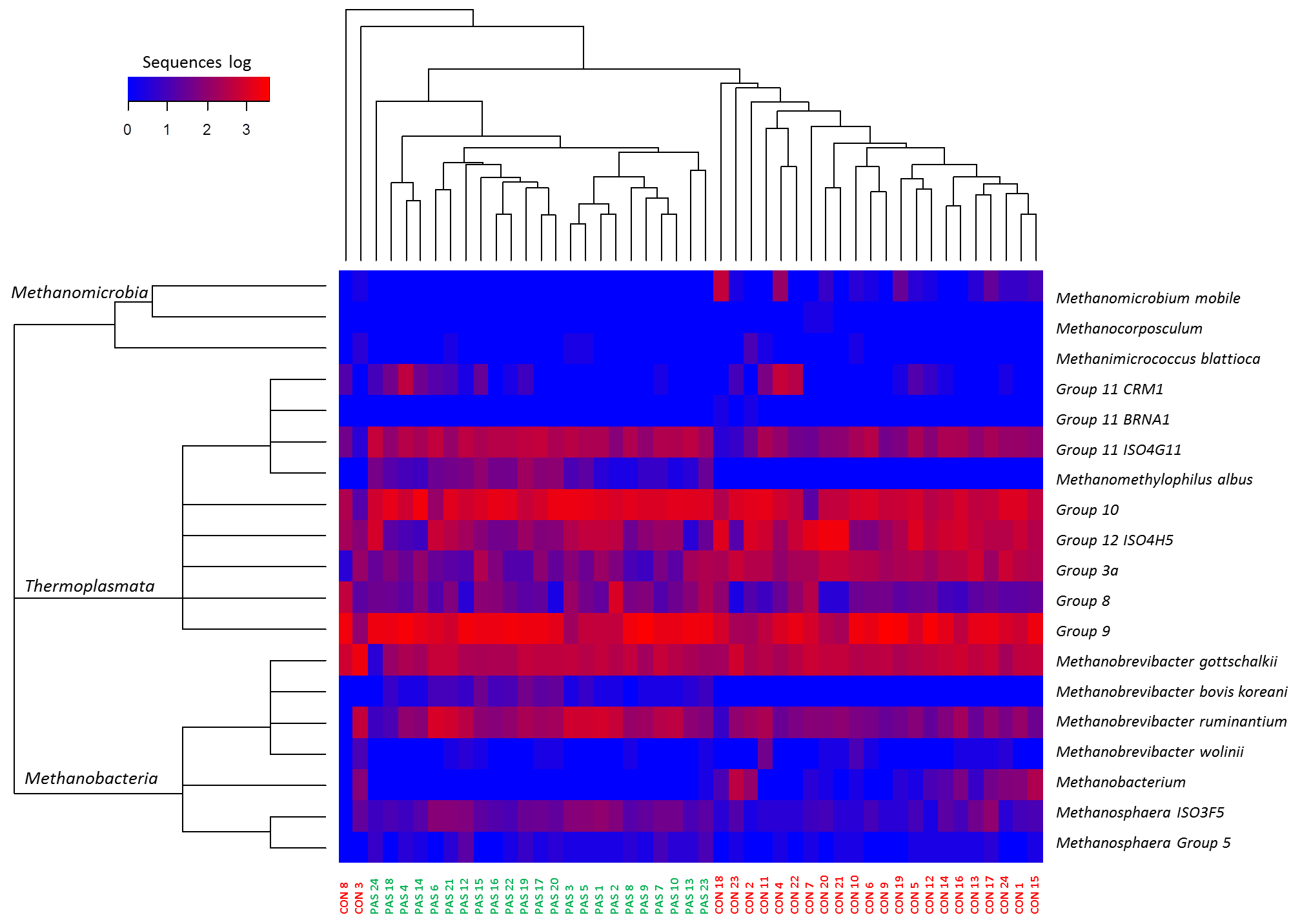


**Figure S2.** Heatmap describing the effect of the diet on the structure of the methanogens community in the rumen of sheep. CON: ryegrass hay diet supplemented with concentrate; PAS; ryegrass pasture. Dendrogram based on the UPGMA clustering of the Bray-Curtis distances. The number of reads per sample was normalized and log transformed.


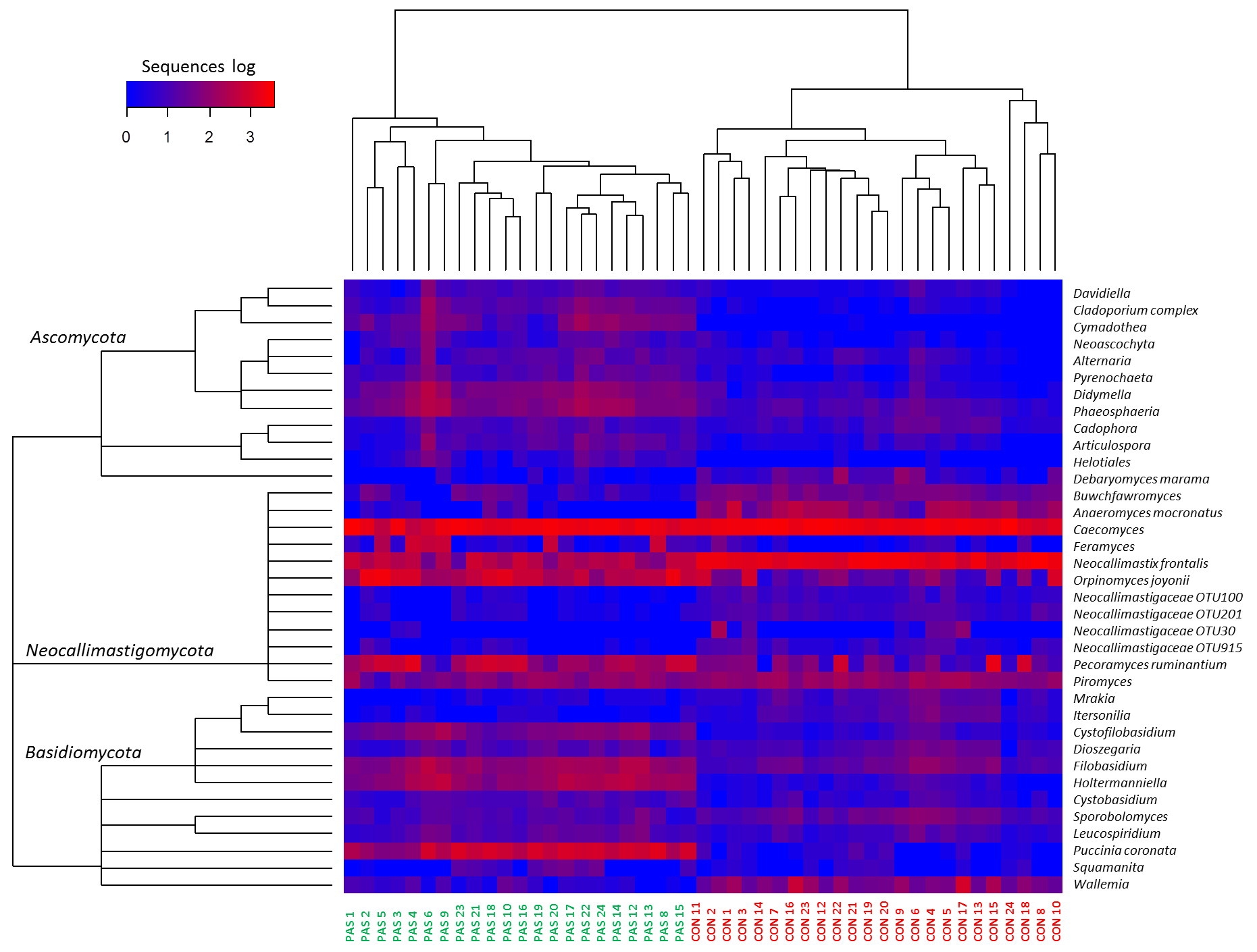


**Figure S3.** Heatmap describing the effect of the diet on the structure of the fungal community in the rumen of sheep. CON: ryegrass hay diet supplemented with concentrate; PAS; ryegrass pasture. Dendrogram based on the UPGMA clustering of the Bray-Curtis distances. The number of reads per sample was normalized, log transformed and minor genera discarded (<0.05%).


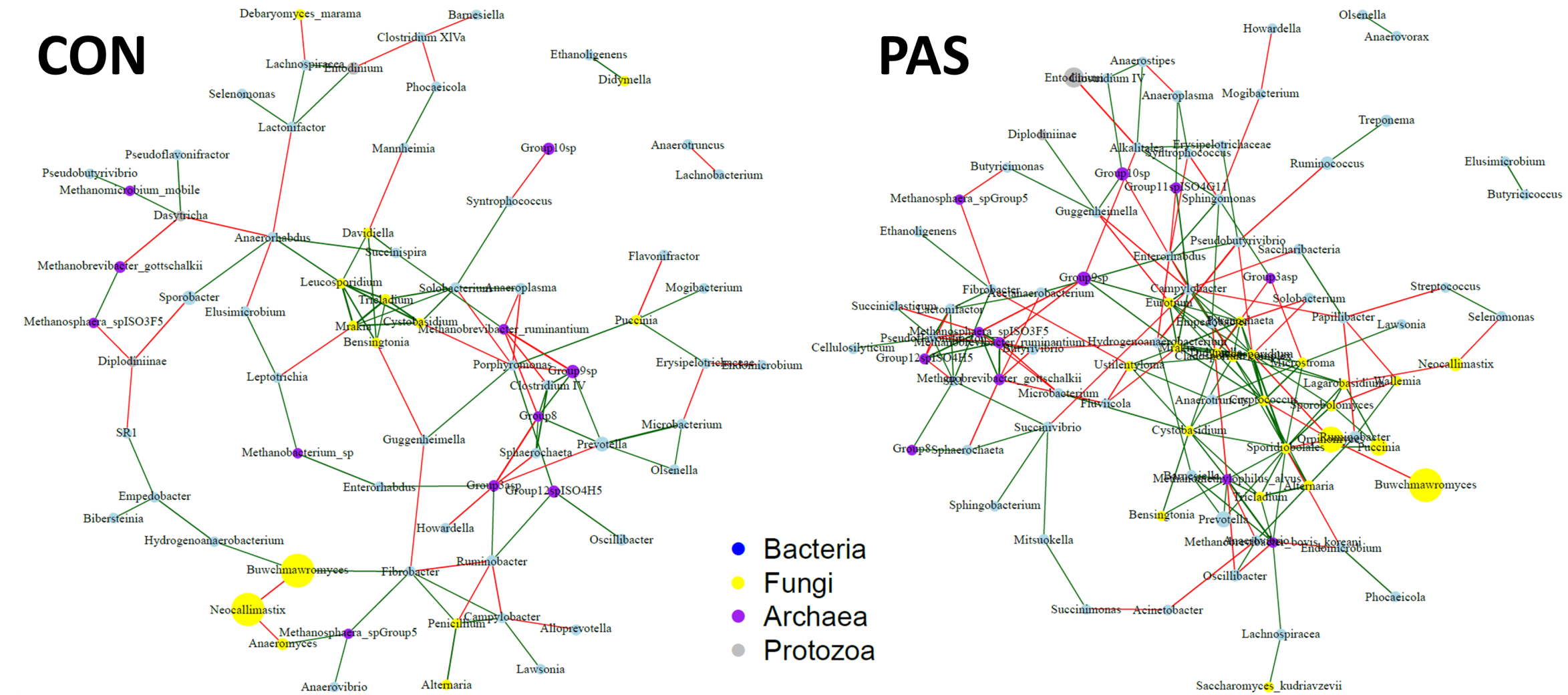


**Figure S4.** Microbial network illustrating the effect of the diet on the relationships between the different microbial groups in the rumen of sheep. Edges represent positive (green) and negative (red) correlation coefficients between taxonomical groups (*r* > 0.5 and adjusted *P*<0.05). CON: ryegrass hay diet supplemented with concentrate; PAS; ryegrass pasture

**References**

Denman, S. E. and C. S. McSweeney. 2006. Development of a real-time PCR assay for monitoring anaerobic fungal and cellulolytic bacterial populations within the rumen. *FEMS Microbiol. Ecol*. **58**:572-582.

Denman, S. E., N. Tomkins, and C. S. McSweeney. 2007. Quantitation and diversity analysis of ruminal methanogenic populations in response to the antimethanogenic compound bromochloromethane. *FEMS Microbiol. Ecol*. **62**:313-322.

Detheridge, A. P., G. Brand, R. Fychan, F. V. Crotty, R. Sanderson, G. W. Griffith, and C. L. Marley. 2016. The legacy effect of cover crops on soil fungal populations in a cereal rotation. *Agric. Ecosyst. Environ*. **228**:49-61.

Maeda, H., C. Fujimoto, Y. Haruki, T. Maeda, S. Kokeguchi, M. Petelin, H. Arai, I. Tanimoto, F. Nishimura, and S. Takashiba. 2003. Quantitative real-time PCR using TaqMan and SYBR Green for *Actinobacillus actinomycetemcomitans, Porphyromonas gingivalis, Prevotella intermedia*, tetQ gene and total bacteria. *FEMS Immunol. Med. Microbiol*. **39**:81–86.

Spear, G. T., M. Sikaroodi, M. R. Zariffard, A. L. Landay, A. L. French, and P. M. Gillevet. 2008. Comparison of the diversity of the vaginal microbiota in HIV-infected and HIV-uninfected women with or without bacterial vaginosis. J.f Infec. Dis. **198**:1131-1140.

Sylvester, J. T., S. K. R. Karnati, Z. T. Yu, M. Morrison, and J. L. Firkins. 2004. Development of an assay to quantify rumen ciliate protozoal biomass in cows using real-time PCR*. J. Nutr*. **134**:3378-3384.

Wright, A. D. G. and C. Pimm. 2003. Improved strategy for presumptive identification of methanogens using 16S riboprinting. *J. Microbiol. Meth*. **55**::337-349
